# Crosslinking of CD38 Receptors Triggers Apoptosis of Malignant B Cells

**DOI:** 10.3390/molecules26154658

**Published:** 2021-07-31

**Authors:** M. Tommy Gambles, Jiahui Li, Jiawei Wang, Douglas Sborov, Jiyuan Yang, Jindřich Kopeček

**Affiliations:** 1 Center for Controlled Chemical Delivery, University of Utah, Salt Lake City, UT 84112, USA; u0480778@utah.edu (M.T.G.); Jiahui.Li@pharm.utah.edu (J.L.); jiawei.wang@utah.edu (J.W.); 2 Department of Pharmaceutics and Pharmaceutical Chemistry, University of Utah, Salt Lake City, UT 84112, USA; 3 Huntsman Cancer Institute, University of Utah, Salt Lake City, UT 84112, USA; Douglas.Sborov@hci.utah.edu; 4 Department of Biomedical Engineering, University of Utah, Salt Lake City, UT 84112, USA

**Keywords:** CD38, drug-free macromolecular therapeutics, human serum albumin conjugates, morpholino oligonucleotides, daratumumab, isatuximab, multiple myeloma, lymphoma

## Abstract

Recently, we designed an inventive paradigm in nanomedicine—drug-free macromolecular therapeutics (DFMT). The ability of DFMT to induce apoptosis is based on biorecognition at cell surface, and crosslinking of receptors without the participation of low molecular weight drugs. The system is composed of two nanoconjugates: a bispecific engager, antibody or Fab’ fragment—morpholino oligonucleotide (MORF1) conjugate; the second nanoconjugate is a multivalent effector, human serum albumin (HSA) decorated with multiple copies of complementary MORF2. Here, we intend to demonstrate that DFMT is a platform that will be effective on other receptors than previously validated CD20. We appraised the impact of daratumumab (DARA)- and isatuximab (ISA)-based DFMT to crosslink CD38 receptors on CD38+ lymphoma (Raji, Daudi) and multiple myeloma cells (RPMI 8226, ANBL-6). The biological properties of DFMTs were determined by flow cytometry, confocal fluorescence microscopy, reactive oxygen species determination, lysosomal enlargement, homotypic cell adhesion, and the hybridization of nanoconjugates. The data revealed that the level of apoptosis induction correlated with CD38 expression, the nanoconjugates meet at the cell surface, mitochondrial signaling pathway is strongly involved, insertion of a flexible spacer in the structure of the macromolecular effector enhances apoptosis, and simultaneous crosslinking of CD38 and CD20 receptors increases apoptosis.

## 1. Introduction

The use of monoclonal antibodies (mAb) in the treatment of hematological malignancies has become an essential part of immunotherapy regimens [1]. Often mAb’s are used in combination with small molecule chemotherapeutics to improve patient prognoses. Immunotherapy offers highly specific targeting to overexpressed cancer cell surface antigens. Once engaged with their target cell surface receptor, various mechanisms of action may occur to initiate cancer cell death. Immune effector cells can interact with the Fc domains leading to a variety of cell death events including complement-dependent cytotoxicity (CDC), antibody-dependent cellular cytotoxicity (ADCC), and antibody-dependent cellular phagocytosis (ADCP) [2]. Additionally, crosslinking of some receptor-bound antibodies leads to a clustering effect of surface receptors that triggers apoptotic mechanisms within the cell.

Crosslinking of cell-surface receptors has important biological consequences, including enhancing internalization of receptor-ligand complexes [3,4], changing the subcellular fate of the receptor-ligand complex from recycling to the lysosomal route [5], and phosphatidylserine translocation and apoptosis initiation [6].

**Scheme 1:**
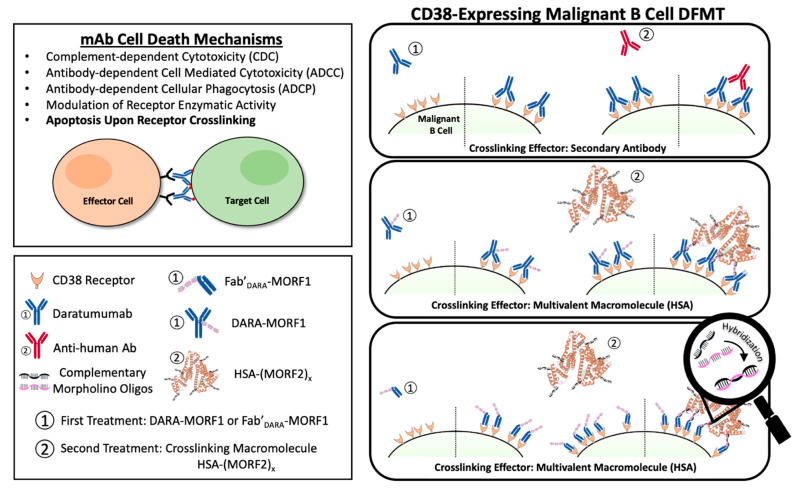
CD38-targeting DFMT system depicted using DARA-MORF1 as the bispecific engager and HSA-(MORF2)_y_ as the crosslinking effector molecule. DFMT is a two-step process involving step (1): specific antigen engagement by the bispecific engager followed by step (2): receptor crosslinking via the effector nanoconjugate. Crosslinking occurs via hybridization of complementary morpholino oligonucleotides conjugated to the bispecific engager and effector molecules.

The CD38 receptor is substantially expressed on multiple myeloma cells and at low levels on normal lymphoid and myeloid cells. It is also expressed on lymphoma cells. In addition to its receptor function, CD38 is an ectoenzyme that cleaves nicotinamide dinucleotide phosphate (NADP) and nicotinamide adenine dinucleotide (NAD^+^) [7]. DARA (fully human IgG1-κ) [8] and ISA (chimeric IgG1-κ) [9] are two FDA approved antibodies for multiple myeloma (MM) treatment [7]. DARA’s mechanisms of action include CDC, ADCC, and ADCP. In addition, FcγR-mediated crosslinking of tumor-bound DARA initiates cell death (Scheme 1) [6]. ISA’s mechanism of action includes CDC, ADCC, and ADCP. Importantly, ISA has a strong apoptosis-inducing activity that is independent of crosslinking and inhibits the enzymatic activity of CD38 [10].

Drug-free macromolecular therapeutics (DFMT) is a new paradigm for the treatment of malignancies [11,12,13]. Induction of apoptosis is mediated by receptor crosslinking facilitated by biorecognition of complementary peptide or oligonucleotide motifs; no low molecular weight drug is needed [11,12]. DFMT is comprised of two complementary nanoconjugates: (a) the bispecific engager: an antibody or Fab’ fragment conjugate with morpholino oligonucleotide MORF1; and (b) the crosslinking effector: *N*-(2-hydroxypropyl)methacrylamide (HPMA) copolymer or human serum albumin (HSA) modified with multiple copies of complementary oligonucleotide MORF2. Hybridization of MORF1/MORF2—mediated receptor crosslinking initiates apoptosis (Scheme 1). We have demonstrated the efficacy of DMFT on CD20 positive (CD20+) Raji B cells in vitro [14,15], in vivo on a disseminated non-Hodgkin lymphoma (NHL) model in SCID mice [16,17], and on patient cells diagnosed with various blood borne malignancies [18]. Apoptosis induction by DMFT is triggered by relocation of crosslinked CD20 complexes to lipid rafts resulting in calcium influx, mitochondrial depolarization, and caspase 3 activation [19].

The linkers used in the DFMT nanoconjugations are based on a bifunctional PEG. Intact nanoconjugates, therefore, have inert linkers consisting of short PEG dimers with amide and thioether bonds on either terminus. All antibodies used in this work are FDA approved products which have overcome much scrutiny in terms of toxicity, pharmacokinetics and biocompatibility [20,21,22]. Human serum albumin is a ubiquitous protein in human plasma and is gaining more and more interest in drug delivery systems because of its long circulation half-life and non-immunogenicity [23]. The morpholino oligomer strands are DNA analogues that have had their backbone chemistry altered to allow for protease resistance in vivo. Numerous oligonucleotides have been evaluated in clinical trials and proven their biocompatibility [24]. A detailed study of the immune properties of the crosslinking effector HSA-(MORF2)x is planned using protocols we developed when evaluating the peptide containing crosslinking effector P-(CCK)x [25].

Rituximab (RTX) and other Type I antibodies dramatically improved treatment of CD20+ B-cell hematological malignancies [26]. However, a number of patients develop resistance and due to polymorphism of Fcγ receptors on immunocompetent cells the hyper-crosslinking of RTX bound to CD20 is not efficient. This catalyzed the advance of Type II antibodies, such as Obinutuzumab (OBN), that do not need crosslinking. They induce apoptosis by actin rearrangement, lysosomal disruption, and homotypic cell adhesion [21].

Interestingly, when DFMT was applied to OBN, classified as a Type II antibody, a system that combines Type I and Type II mechanisms was developed [27]. The first nanoconjugate was OBN-MORF1 (OBN conjugated to one morpholino oligonucleotide MORF1); and the second nanoconjugate was HSA-(MORF2)_y_ (HSA grafted with multiple copies of complementary morpholino oligonucleotide 2). Modification of OBN with one MORF1 does not impact the binding of OBN-MORF1 to CD20 and following binding to CD20 Type II effects occur. Further exposure to multivalent effector HSA-(MORF2)_y_ results in crosslinking of CD20-OBN-MORF1 complexes, their clustering into lipid rafts and initiation of Type I effects. This new approach, called “clustered OBN (cOBN)” combines effects of both cell death-inducing mechanisms resulting in very high apoptotic levels [27].

Aiming to improve the efficacy of treatment of B cell malignancies in general and of multiple myeloma in particular, we evaluated the impact of crosslinking CD38 receptors on the mechanism and extent of apoptotic induction in four CD38 positive malignant B cells (Daudi, Raji, RPMI 8226, and ANBL-6). DFMT based on Fab’_DARA_-MORF1, Fab’_ISA_-MORF1, DARA-MORF1, and ISA-MORF1 as bispecific engagers and HSA-PEG_x_-(MORF2)_y_ as multivalent crosslinking effector were evaluated. The ultimate goal of our studies is to establish the cell and nanoconjugate structure-dependent participation of Type I and Type II apoptotic mechanisms in CD38 crosslinking-mediated apoptosis induction.

## 2. Results and Discussion

### 2.1. Nanoconjugates Synthesis and Cell Lines

**Figure 1:**
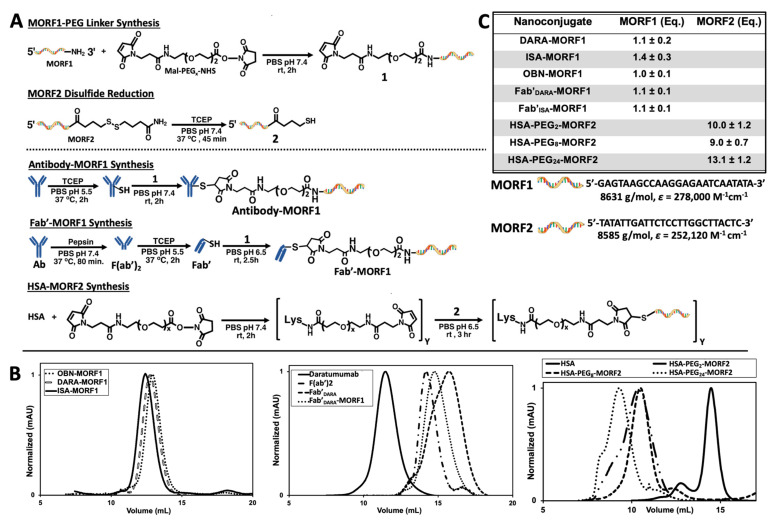
Nanoconjugate synthesis and characterization. (**A**) Synthetic route to produce antibody-MORF1, Fab’-MORF1 and HSA-(MORF2)_y_ conjugates. Note: If not otherwise stated all conjugates contain a diethyleneglycol unit in the spacer inserted by SM-PEG2. Conjugates containing longer spacers are denoted as, e.g., Fab’-PEG_8_-MORF1. (**B**) Nanoconjugate size exclusion chromatography profiles detected on a Superdex 200 10/300 GL column, PBS (pH 7.4) as eluant at 0.4 mL/min flow rate. (**C**) Characterization (MORF valence) of nanoconjugates determined using UV-Vis absorbance at λ = 260 nm and BCA assay.

DARA and ISA nanoconjugates were synthesized using procedures previously reported by our group (Figure 1A) [27,28,29]. First, whole antibody was selectively reduced targeting the interchain disulfide bonds located in the hinge region. In parallel to the reduction reaction, the 3′-amine-functionalized MORF1 was reacted with maleimide-PEG_2_-NHS by aminolysis, resulting in maleimide-MORF1 intermediate. The latter was then coupled with freshly reduced whole antibody via thiol–ene click chemistry yielding the desired antibody-MORF1 nanoconjugate. The Fab’ fragment MORF1 analogues were generated by first digesting whole antibody with pepsin resulting in dual chain cleavage below the disulfide hinge region generating the divalent F(ab’)_2_ intermediate. F(ab’)_2_ was then reduced to generate two equivalents of Fab’ fragments which were further coupled via a thiol–ene click reaction with the maleimide-MORF1 intermediate yielding the desired Fab’-MORF1 nanoconjugate. The HSA-(PEG)_x_-(MORF2)_y_ nanoconjugate was synthesized in a similar fashion utilizing the bifunctional reactivity of maleimide-(PEG)_x_-NHS (SM-PEG). Free lysine amine groups on the periphery of the HSA molecule were coupled with maleimide-PEG_x_-NHS (x = 2, 8, or 24) yielding the multivalent maleimide functionalized HSA-PEG_x_-maleimide intermediate. The complementary morpholino, MORF2, was customized with an easily reducible disulfide bond on its 3′ terminus. HSA-PEG_x_-maleimide was decorated with freshly reduced MORF2 molecules via thiol–ene maleimide click reactions. A valency greater than about 5 in HSA-(MORF2)_y_ did not show significant increase in efficacy of CD20 receptor crosslinking [27]; so minor variations in valency (Figure 1C) should not have an impact on efficacy. Reaction intermediates and final nanoconjugates were characterized by size exclusion chromatography (SEC) (Figure 1B). Each nanoconjugate morpholino valency was characterized by UV-Vis spectrophotometry (Figure 1C) and BCA assay. The valence of MORF per macromolecule was calculated by (i) attaining the concentration of MORF in solution by UV-Vis spectrophotometry and (ii) determining concentration of protein in solution by BCA assay, then (iii) dividing the two concentrations yielding ratio of MORF per molecule. Hybridization between complementary nanoconjugates was confirmed by UV-Vis spectrophotometry by observing absorbance changes at λ = 260 nm of varying molar ratio solutions of MORF1:MORF2 (Figures S2 and S5); and size exclusion chromatography (Figures S1 and S4).

**Figure 2:**
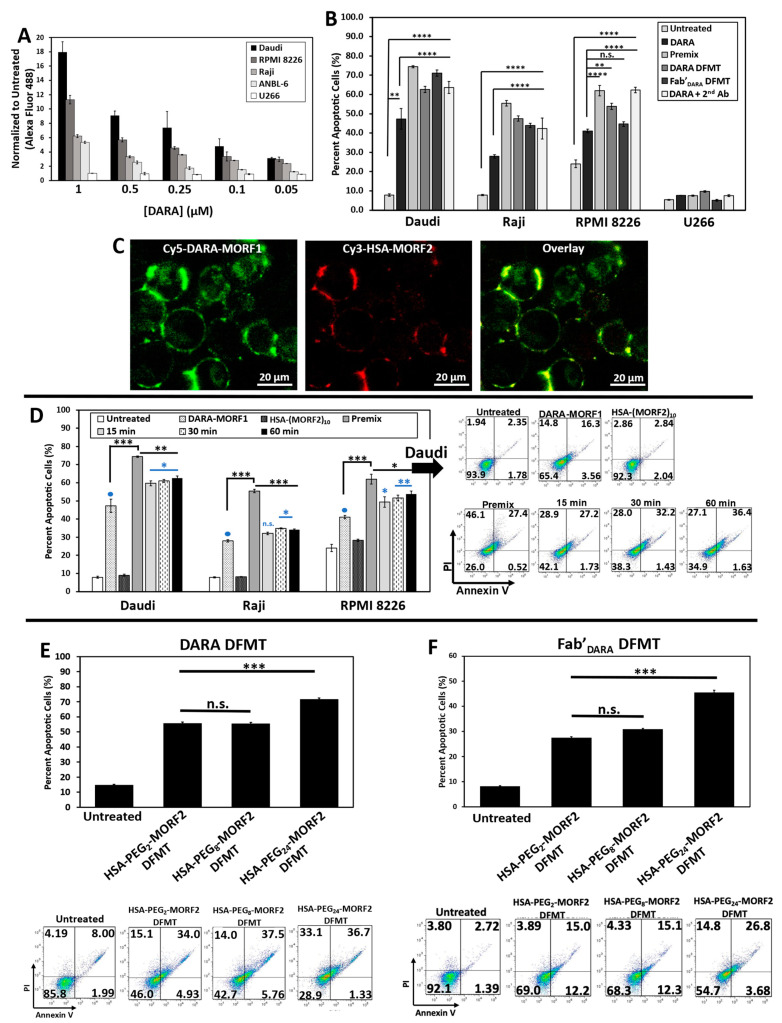
(**A**) DARA binding on various B cell lines as determined by secondary fluorescence of a Fluor 488-labeled anti-human goat antibody. (**B**) Apoptosis induced by DARA-MORF1 and Fab’_Dara_-MORF1 DFMT treatments on various CD38+ cell lines and one CD38- cell line: U266. Cell viability was measured by Annexin V and Propidium Iodide staining and analyzed by flow cytometry. (**C**) Confocal microscopy of fluorescently labeled nanoconjugates: Cy5-DARA-MORF1 (left, green); Cy3-HSA-(MORF2)_10_ (middle, red); overlay (right, yellow) of Cy5 and Cy3 channels on Daudi cells. (**D**) Effect of time lag (15, 30, and 60 min) between the administration of the bifunctional engager, DARA-MORF1 and the multivalent effector, HSA-(MORF2)_10_ on apoptosis initiation. (**E**,**F**) Effect of HSA PEG linker length on DFMT apoptosis efficacy on Daudi cells. (**E**) DARA-MORF1 + HSA-PEG_2,8,24_-(MORF2)_y_. (**F**) Fab’_DARA_-MORF1 + HSA-PEG_2,8,24_-(MORF2)_y_. Flow cytometry cell population distribution data for the HSA PEG linker length studies are also shown. All experiments were performed in triplicate. **** *p* < 0.0001, *** *p* < 0.001, ** *p* < 0.01, * *p* < 0.05, n.s., not significant by One-Way ANOVA and Tukey test.

Four CD38 positive cell lines were used: Raji (Burkitt’s lymphoma), Daudi (Burkitt’s lymphoma), RPMI 8226 (multiple myeloma), and ANBL-6 (multiple myeloma). CD38 negative cell line U266 (multiple myeloma) was used as control. Level of CD38 expression was estimated by DARA binding to each cell line at 4 °C followed by exposure to a fluorescently labeled anti-human secondary antibody. The level of CD38 expression was Daudi > RPMI 8226 > Raji > ANBL-6 >> U266 (Figure 2A).

### 2.2. DFMT Triggers Apoptosis in CD38+ Lymphoma and Myeloma Cell Lines by Consecutive Binding of Nanoconjugates

To validate the hypothesis that crosslinking of CD38 directly initiates apoptosis, we evaluated the levels of apoptosis initiation in Daudi, Raji, RPMI 8226, ANBL-6, and U266 cell lines by exposing them to DARA-MORF1 or Fab’_DARA_-MORF1 (0.5 µM MORF1) for 1 h, followed (after washing and resuspending) to HSA-(MORF2)_10_ (0.5 µM MORF2) for 24 h. High levels of apoptosis were achieved in the three CD38+ cell lines (Daudi cells exhibited the highest levels) as well as in controls, premix and Daratumumab + sec. antibody. As expected, CD38- U266 cells exhibited negligible levels of apoptosis. Interestingly, percentage of apoptotic cells for the various cell types correlated with the level of CD38 expression observed in the binding studies (Figure 2A,B).

We next investigated the biorecognition of nanoconjugates at the cell surface employing confocal fluorescence microscopy. Consecutive exposure of Raji cells to Cy5-DARA-MORF1 resulted in cell surface green signal; exposure of decorated cells to HSA-(MORF2)_10_ showed red surface signal. Both signals were colocalized (yellow color) indicating successful biorecognition (hybridization) of MORF1/MORF2 at cell surface (Figure 2C).

DFMT is a two-step process: The first nanoconjugate a bispecific engager, DARA-MORF1 or Fab’_DARA_-MORF1, binds to CD38 and decorates the cell surface with MORF1 moieties. After a time lag, the second nanoconjugate, a multivalent macromolecular effector, HSA-PEG_x_-(MORF2)_y,_ hybridizes and crosslinks multiple CD38 receptors resulting in apoptotic response. One important factor related to the efficacy of the process is the potential internalization of CD38 following binding with the bispecific engager. It is known that surface CD38 is internalized after receptor binding [30,31]. The internalization is gradual with time and crosslinking enhances the rate of internalization on the Jurkat cell line [30]. To validate the two-step pretargeting approach, we compared apoptosis induction for different time lags between cells’ (Raji, Daudi, and RPMI 8226) exposure to the two nanoconjugates; the second nanoconjugate HSA-(MORF2)_10_ was administered after 15 min, 30 min, and 1 h after the administration of the bispecific engager (Figure 2D and Figure S7). Additionally, we exposed cells to a multivalent premix of both conjugates (control). In all three cell lines the length of the time lag had no impact on the level of apoptosis. Premixing nanoconjugates before cell exposure enhanced apoptotic levels when compared to two-step administration. The difference was largest in Raji cells and minor in Daudi and RPMI 8226 cells. This may be the effect of crosslinking enhanced internalization of the loaded CD38 receptor. The difference in apoptosis induction between premixed nanoconjugates and consecutive administration was minimal for the CD20 receptor [28], reflecting different internalization kinetics of CD20 vs. CD38 following receptor binding and crosslinking.

We described the advantages of the two-step administration previously, e.g., [32]. Importantly, a two-step approach permits pretargeting in vivo, a strategy commonly used in cancer radioimmunotherapy [33,34]. The experiments in this work were performed in vitro which makes the nanoconjugate premixture a meaningful control treatment group because hybridization is allowed to occur in an idealized setting and no washing step between treatments is needed. This provides a theoretical “maximum efficacy” for the in vitro experiments. For in vivo applications, one must consider important factors such as immune response, effector cell interactions and clearance and how each of these factors influence both the targeting of the system and the hybridization.

Pretargeting strategy (two-step treatment) permits the amplification of therapeutic efficacies and reduces adverse side reactions [35]. For example, in our previous work with DFMT targeted to CD20 we determined the time lag when the pretargeting agent (Fab’-MORF1) was mostly cleared from the blood and reached a steady plasma concentration, and, second, by determining the tumor targeting efficiency when using this time interval [14]. Results indicated a suitable timing for P-(MORF2)_x_ administration at 5 h (in female SCID mice); at this time, Fab’-MORF1 was efficiently distributed to the tumors. Based on this result, we further performed therapy experiments in a disseminated B-NHL mouse model. When the optimized pretargeting time lag (5 h) was used, the therapeutic efficacy was significantly better than that of identical experimental conditions but with a 1 h interval. A low dose (58 μg × 3) of Fab’-MORF1 with a 5× excess P-(MORF2)_x_ resulted in significantly delayed tumor growth and substantially improved animal survival [14]. The optimized therapeutic system surpassed rituximab in anticancer efficacy and completely eradicated lymphoma B-cells in 83% of the animals. This pretargeting approach may constitute a novel personalized nanotherapy to enable more efficient treatment and limit potential side effects associated with off-target binding.

The decreased adverse (off-target) effects of two-step administration in vivo are based on these phenomena: CD20 is a very slowly internalizing receptor. When Fab’-MORF1 is administered, the part bound to CD20 remains at the surface, whereas the off-target bound part is internalized (and degraded in the lysosomes) during the time-lag before the administration of the crosslinking effector. Thus, the crosslinking effector administered following a time-lag, finds the bispecific engager bound just to the target (CD20).

### 2.3. Impact of Spacer Length on Apoptosis Initiation

Spacer length is an important factor in the efficiency of nanoconjugates. PEG spacers enhanced the efficacy of DMFT systems based on flexible HPMA copolymer molecules [28] as well as multivalent liposomes [36]. Since HSA has a relatively rigid structure, introduction of a flexible spacer between HSA and MORF2 should enhance biorecognition and apoptosis induction. We used succinimidyl-PEG_x_-maleimides, hetero-bifunctional crosslinkers with different numbers of repeating ethyleneglycol (EG) units to synthesize HSA-PEG_x_-MORF2 conjugates with variable spacer length. In particular, NHS-PEG_2_-maleimide (succinimidyl-[(*N*-maleimidopropionamido)-diethyleneglycol]ester), NHS-PEG_8_-maleimide (succinimidyl-[(*N*-maleimidopropionamido)-octaethyleneglycol] ester), and NHS-PEG_24_-maleimide (succinimidyl-[(*N*-maleimidopropionamido)-tetracosaethylene glycol] ester) (Thermo Scientific) were used for the synthesis of HSA-PEG_2_-(MORF2)_10_, HSA-PEG_8_-(MORF2)_9_, and HSA-PEG_24_-(MORF2)_13_. The characterization of conjugates is shown in Figure 1b (*right panel*). Apoptosis was determined on Raji cells by Annexin V/PI assay. Two bispecific engagers, DARA-MORF1 and Fab’_DARA_-MORF1 were employed. Data in Figure 2E,F show no statistically significant difference between spacers containing PEG_2_ (17.6 Å) and PEG_8_ (39.2 Å). Increasing the length of the spacer to PEG_24_ (95.2 Å) resulted in statistically significant enhancement of apoptosis induction. This is valid for both bispecific engagers used. It appears that a relatively rigid carrier, such as HSA, needs a longer spacer to enhance efficacy when compared to a flexible HPMA copolymer carrier. In the latter DFMT system (Fab’-MORF1 + HPMA copolymer-MORF2) a statistically significant enhancement of apoptosis was observed when increasing the spacer length form PEG_2_ to PEG_8_ [28].

### 2.4. Prevention of Calcium Influx and Cholesterol Depletion from Lipid Rafts Lessen Apoptosis

Two important features were observed when initiating CD20 mediated apoptosis by DFMT. Crosslinking of CD20 receptors in Raji cells resulted in rapid rise in the Ca++ intracellular concentration [19]. Additionally, extracting cholesterol from cell membranes by β-cyclodextrin (β-CD) impacted receptor clustering as detected by STORM (stochastic optical reconstruction microscopy) [15]. Cholesterol is an important part of lipid rafts and contributes to mechanisms of anti-CD20 antibodies action [37,38]. Both phenomena seem to be correlated; transfer of loaded CD20 into lipid rafts promotes calcium influx [39].

**Figure 3:**
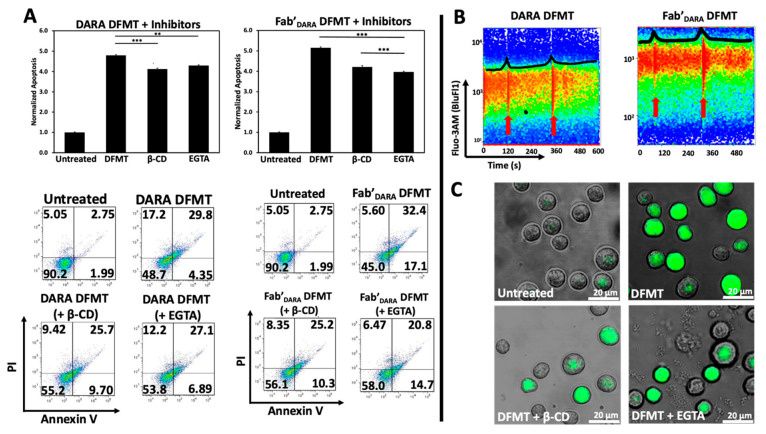
(**A**) Investigation of Raji cell apoptosis induced by DFMT with or without pretreatment with 0.02 wt% β-CD or 1 mM EGTA. (**B**) Flow cytometry time lapse fluorescence of calcium chelator Fluo-3AM after addition of nanoconjugates in DARA-based DFMT (DARA-MORF1 + HSA-(MORF2)_10_ (left panel) and Fab’_DARA_-based DFMT (Fab’_DARA_-MORF1 + HSA-(MORF2)_10_) (right panel). Red arrows indicate time of nanoconjugate addition to cell sample. (**C**) Confocal microscopy images of β-CD or EGTA treated Daudi cells compared to normal cells undergoing Fab’_DARA_-based DFMT. *** *p* < 0.001, ** *p* < 0.01, n.s. nonsignificant, by One-Way ANOVA and Tukey test.

We hypothesized that crosslinking of CD38 receptors by DFMT will have similar impact on the apoptosis initiation as observed with CD20 receptors. To this end, we preincubated Raji cells either with 0.02% β-CD (to extract cholesterol) or with 1 mM EGTA (to chelate extracellular calcium) before exposing them to DFMT. Following pretreatment, decrease in apoptotic levels was observed for both, DARA-based DFMT (DARA-MORF1 followed 1 h later by HSA-(MORF2)_10_) and Fab’_DARA_-based DFMT (Fab’_DARA_-MORF1 followed 1 h later by HSA-(MORF2)_10_). Data seem to suggest a higher impact of pretreatment on Fab’_DARA_-based DFMT. When normalized to untreated, the percent apoptotic cells pretreated with β-CD decreased by 0.7-folds for DARA-based DFMT and by about 1.0-folds for Fab’_DARA_-based DFMT. The percent apoptotic cells pretreated with EGTA decreased by 0.5-folds for DARA-based DFMT and by 1.2-folds for Fab’_DARA_-based DFMT (Figure 3A).

Calcium influx was also measured directly by using a fluorescent calcium chelator, Fluo-3AM, to observe calcium influx into Raji cells following DARA-based DFMT and Fab’_DARA_-based DFMT (Figure 3B). Raji cells were used because the cell line is both CD20 and CD38 positive. Therefore, we could compare CD38-induced calcium influx with previously reported CD20-induced calcium influx data [19,27]. Red arrows indicate the time at which the nanoconjugates were added to the cell samples (Figure 3B). The DARA-MORF1 treatment had a distinct calcium signal spike immediately upon addition to the sample; however, the Fab’_DARA_-MORF1 had a much less prominent Ca^2+^ influx event upon addition of the Fab’ nanoconjugate. Conversely, upon addition of HSA-(MORF2)_10_ to both samples, the calcium spike was more pronounced for the Fab’_DARA_-based DFMT than whole antibody-based DFMT corresponding with the calcium inhibition data (Figure 3B). As mentioned above, partial inhibition of calcium influx impacted the efficacy of Fab’_DARA_-based DFMT more than it impacted DARA-based DFMT. The calcium influx observed in Fab’_DARA_-based DFMT treated cells corresponds with the results of the calcium inhibition experiments where calcium inhibition hampered the Fab’_DARA_-based DFMT efficacy over DARA-based DFMT. The inhibition of calcium influx was also confirmed by confocal fluorescence microscopy (Figure 3C). Fluorescence was markedly lower in the EGTA and β-CD pretreated cells. Raji cells are CD38+/CD20+ so comparison of effects resulting from crosslinking of both receptors seem to indicate a stronger response following crosslinking of CD20 (comparing data of this manuscript with [19,27]).

### 2.5. DARA- and Fab’_DARA_-based DFMT Induce Apoptosis via Mitochondrial Signaling Pathway

We next investigated the possible activation of the mitochondrial signaling pathway following crosslinking of decorated CD38 receptors on Daudi cells by multivalent macromolecular effector. The major features of the mitochondrial pathway include mitochondrial depolarization, cytochrome C release, caspase 3 activation, and bcl-2 downregulation [19,40].

**Figure 4:**
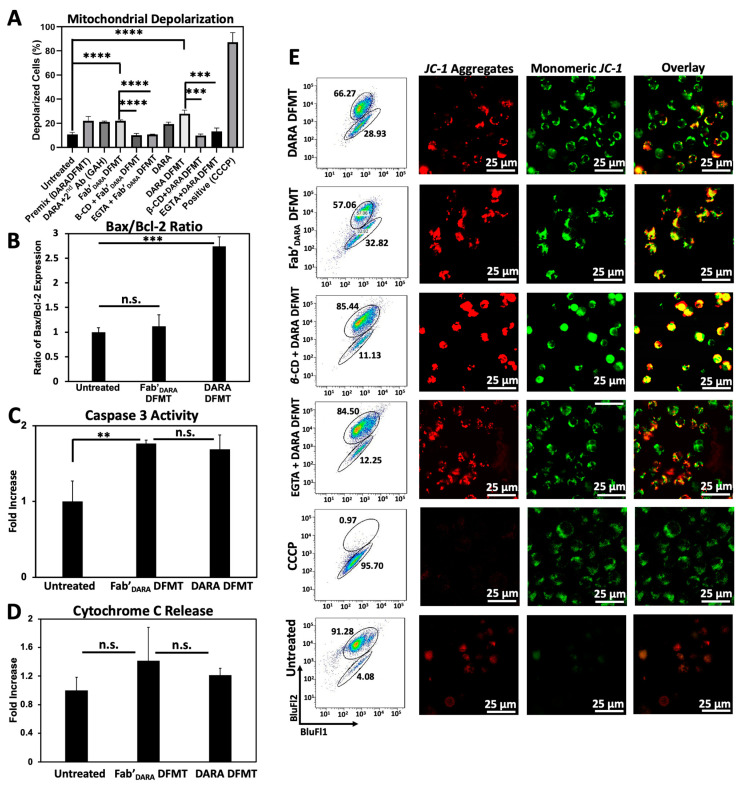
Investigation of mitochondrial pathway involvement in apoptosis induction in Daudi cells. (**A**) Compiled mitochondrial membrane depolarization of DARA DFMT and Fab’_DARA_ DFMT treated Daudi cells with or without the presence of inhibitors (EGTA or β-CD). Controls include untreated cells, a DARA-based DFMT “Premix” group, DARA crosslinked with a secondary antibody (goat antihuman: GAH), and a positive control treatment with CCCP. Depolarization was measured by JC-1 red/green fluorescence by flow cytometry. (**B**) Bax/Bcl-2 expression ratio of DARA-based DFMT and Fab’_DARA_-based DFMT treated Daudi cells. Expression was measured using immunostaining and flow cytometry. (**C**) Caspase 3 activity of DARA DFMT and Fab’_DARA_ DFMT treated Daudi cells was measured using PhiPhiLux^®^ assay kit and flow cytometry. (**D**) Cytochrome C release of DARA and Fab’_DARA_ DFMT on Daudi cells. Increase in Cytochrome C release shown as fold increase over untreated cells. Determined by ELISA. (**E**) Confocal fluorescence microscopy visualization of mitochondrial depolarization of DARA DFMT with or without the presence of EGTA or β-CD. More red fluorescence indicates healthier mitochondria while more green fluorescence indicates the occurrence of depolarization. **** *p* < 0.0001, *** *p* < 0.001, ** *p* < 0.01, n.s. nonsignificant, by One-Way ANOVA and Tukey test.

Mitochondrial depolarization was assayed using the JC-1 mitochondrial membrane polarization sensor. In healthy mitochondria, membrane polarization remains intact and JC-1 aggregation occurs resulting in red fluorescence emission. As membrane potential diminishes, JC-1 can diffuse out of the mitochondria, thereby losing its red fluorescent signature as aggregates disperse into monomers. This solubilization event is observed by a change in fluorescent signature from red to green fluorescence. Therefore, red fluorescence indicates healthy mitochondria while green fluorescence indicates depolarized mitochondrial membranes. Mitochondrial membrane potential for DARA-based DFMT and Fab’_DARA_-based DFMT was investigated using flow cytometry (Figure 4A) and confocal microscopy (Figure 4D) with and without the presence of EGTA and β-CD. The amount of observed mitochondrial depolarization was larger for DARA-based DFMT than Fab’_DARA_-based DFMT, but both had higher mitochondrial membrane depolarization than DARA alone. This higher mitochondrial membrane depolarization observed in DFMT-treated cells compared to naked mAb is consistent with the higher apoptosis observed in the cell viability experiments.

B-cell lymphoma 2 (Bcl-2) and Bcl-2-associated X protein (Bax) expression levels were assayed by fluorescent immunostaining (Figure 4B). DFMT treated or untreated Daudi cells were incubated with fluorescently labeled antibodies specific to these two proteins: Bcl-2 mAb (100) Alexa Fluor^®^ 488 and Bax mAb (2D2) Alexa Fluor^®^ 647. Bcl-2 is located in the outer mitochondrial membrane and inhibits actions of pro-apoptotic proteins such as Bax. The expression level ratio of Bax to Bcl-2 is often used to indicate apoptotic states of cells [41,42]. DARA-based DFMT and Fab’_DARA_-based DFMT treated cells were tested against one another and against untreated cells for Bcl-2/Bax expression. The enhanced Bax/Bcl-2 ratio, especially in DARA-based DMFT (Figure 4B) is the indication of mitochondrial signaling pathway involvement, as supported by data on cytochrome C release (Figure 4D) and caspase 3 activity (Figure 4C). Both, DARA-based DFMT and Fab’_DARA_-based DFMT treated cells demonstrated 150–200% of caspase 3 activity when compared to untreated cells. The enhancement of activity was similar for both DFMT approaches. Apparently, the release of cytochrome C initiates the formation of the apoptosome with procaspase 9 and Apaf-1, followed by activation of procaspase 3 and the activation of the caspase cascade results in cell death [40,43].

### 2.6. Reactive Oxygen Species (ROS) Generation, Lysosomal Enlargement, Translocation to Lipid Rafts

ROS are generated by both Type I and Type II antibodies. In the mechanism of CD20 apoptosis initiation there is a clear distinction of mechanisms between Type I Abs, such as RTX [19] and Type II Abs, such as OBN [44]. Following receptor crosslinking, Type I antibodies induce apoptosis by receptor crosslinking followed by calcium influx, mitochondrial depolarization, and caspase activation. In contrast, Type II antibodies do not need crosslinking, they initiate apoptosis by actin remodeling, homotypic cell adhesion and lysosome disruption. Both types produce ROS.

ISA binds to an epitope independent to that of DARA and provides more enzymatic inhibition of the CD38 function [22]. ISA can induce apoptosis without crosslinking, however, lysosomal breakage and cathepsin B leakage was observed [45]. Initiation of direct apoptosis by DARA upon crosslinking and by ISA without crosslinking was reported in refs. [6,22,45]. In contrast, Moreno et al. did not observe it [46]. The discrepancy was explained by the level of CD38 expression—use of cells transduced with CD38 vs. cells with lower CD38 expression levels close to those in MM patients.

To compare mechanisms of DFMT based on CD38-targeting antibodies, DARA and ISA, with CD20-targeting OBN, Raji cells positive for both receptors were used. Three bispecific engagers, DARA-MORF1, ISA-MORF1, and OBN-MORF1, were employed. Following binding to the corresponding receptor, HSA-(MORF2)_10_ was used for crosslinking.

**Figure 5:**
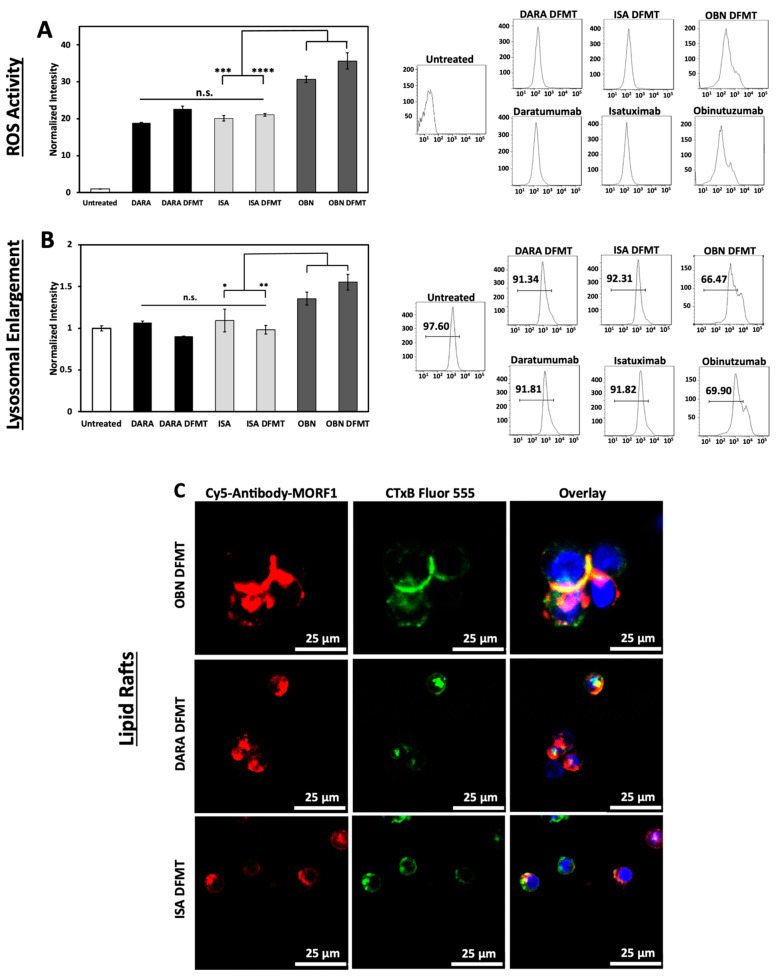
(**A**) Generation of ROS in DARA-, ISA-, and OBN-based DFMT treated Raji cells. The ROS production was measured by oxidation of 2′,7′-dichlorodihydrofluorescein diacetate (H_2_DCFDA) and quantified with flow cytometry. (**B**) Lysosomal enlargement of DARA-, ISA-, and OBN-based DFMT was quantified by LysoTracker Green DND-26 and flow cytometry. (**C**) Confocal microscopy was employed to observe any redistribution of CD38 receptors into cholesterol-rich lipid rafts. Ab-MORF1 (red); lipid raft marker: cholera toxin subunit B (CTxB) Alexa Fluor 555 (green). Nuclei were stained with Hoechst (blue). **** *p* < 0.0001, *** *p* < 0.001, ** *p* < 0.01, * *p* < 0.05, n.s. nonsignificant by One-Way ANOVA and Tukey test.

The production of ROS by DARA, ISA, DARA-based and ISA-based DFMT was at the same level without statistically significant differences (Figure 5A). However, OBN and OBN-based DFMT produced considerably higher amounts of ROS. The highest ROS production was observed in OBN-based DFMT, an indication that both mechanisms (Type I and II) of apoptosis induction are operative.

OBN DFMT showed 1.5-fold increase in lysosome size compared to untreated cells (Figure 5B). Lysosomal enlargement and increased reactive oxygen species are indicative of Type II and coincides with data our group published previously [27].

Additionally, confocal microscopy was employed to investigate redistribution of receptor-bound nanoconjugates on the cell surface into lipid rafts (Figure 5C). Fluorescently labeled antibody-MORF1 conjugates and immunostaining of lipid compartments on the cell surface with cholera toxin subunit B (CTxB) were used to visualize clustering or aggregation of antibody-bound receptors on the cell membrane. Only OBN-based DFMT exhibited typical Type II behavior of intercell homotypic adhesion and lipid raft distribution at cell–cell adhesion sites (Figure 5C *top row*).

DARA-based and ISA-based DFMT treated cells showed no significant increase in lysosome size compared to untreated cells and about only half as much ROS production as OBN-based DFMT treated cells. The confocal imaging showed minimal homotypic cell–cell adhesion and even when some cell adhesion was observed, no pronounced lipid raft accumulation at cell adhesion sites was distinguishable. All of these results are consistent with Type I antibody characteristics for DARA- and ISA-based DFMT. Only OBN-based DFMT demonstrated Type II mechanism characteristics.

The purpose of the ROS assay, the lysosomal enlargement study and the homotypic cell adhesion experiments was to elucidate any Type II antibody mechanisms of action of ISA and ISA-based DFMT. DARA (Type I antibody) and DARA-based DFMT, OBN (Type II antibody) and OBN-based DFMT were used to compare and contrast ISA’s apoptosis induction mechanisms. Type II antibodies are characterized by lysosomal disruption, ROS production and homotypic cell adhesion. Type II antibodies, like OBN, induce apoptosis upon binding to their receptor epitope on the cell surface without the need for crosslinking. It is currently unknown if ISA behaves as a Type II antibody; however, ISA has been shown to induce apoptosis without the need for crosslinking [10]. Therefore, we hypothesized ISA could induce similar lysosomal disruption, ROS production and homotypic cell adhesion as OBN. Our investigation proved otherwise. ISA and ISA-based DFMT did not increase ROS, did not enlarge the lysosomes, nor did we observe any homotypic cell adhesion under confocal microscopy.

**Figure 6:**
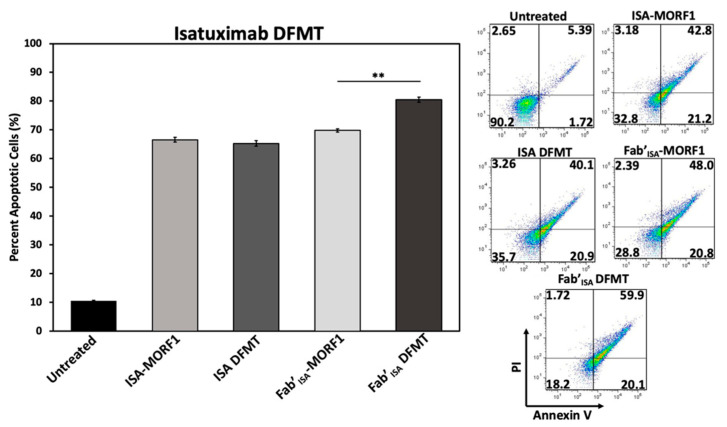
ISA-based DFMT performed on Daudi cells. Apoptosis was measured after ISA DFMT or Fab’_ISA_ DFMT using Annexin V and Propidium Iodide staining. Flow cytometry was performed to assess percentage of apoptotic cells compared to an untreated sample group. Experiment was performed in triplicate and statistically analyzed by One-Way ANOVA and Tukey test. ** *p* < 0.01.

To investigate ISA-based DFMT further, cellular apoptosis experiments were conducted on Daudi cells (Figure 6) and Raji cells (Figure S18) to examine CD38 receptor crosslinking effects when using ISA nanoconjugates. Under the same DFMT cell apoptosis treatment conditions as used for DARA DFMT (Figure 1B), ISA DFMT showed several key differences. First, the same concentration of ISA antibody induced roughly 20 percent more apoptosis than DARA antibody. This could indicate the ISA-CD38 binding epitope to inhibit CD38 enzymatic activity to a larger extent over DARA-CD38 binding, and/or binding of ISA stimulates apoptotic pathways within the cell to a larger extent. Second, Fab’_ISA_-MORF1 control samples showed levels of apoptosis comparable with whole antibody. This is drastically different from Fab’_DARA_-MORF1 controls that showed little to no apoptotic efficacy (Figure S6). Binding of either whole ISA antibody or Fab’ fragment at the ISA binding site induces the therapeutic effect. Lastly, ISA DFMT did not enhance the overall apoptotic efficacy on either Daudi or Raji cells over antibody alone. However, Fab’_ISA_ DFMT did produce significantly increased apoptosis over Fab’_ISA_-MORF1 control and whole antibody.

The findings with ISA suggest antibodies capable of triggering apoptosis directly upon binding to their target antigen will do so regardless of secondary crosslinking. However, the Fab’ fragment crosslinking of this variety of antibody could lead to enhanced efficacy over antibody alone.

### 2.7. Simultaneous Crosslinking of CD38 and CD20 Receptors Enhances Apoptosis

Raji cells are CD38+/CD20+ and crosslinking of both receptors initiates apoptosis. We investigated if dual crosslinking with combination of DFMT based on Fab’ fragments from DARA and RTX would enhance the efficacy of apoptosis induction. To this end we exposed Raji cells to three DFMT treatments (concentrations relate to MORF): (a) 0.5 µM Fab’_DARA_-MORF1 + (1 h later) HSA-(MORF2)_10_; (b) 0.5 µM Fab’_RTX_-MORF1 + (1 h later) HSA-(MORF2)_10_; (c) combination of 0.25 µM Fab’_DARA_-MORF1 + (1 h later) HSA-(MORF2)_10_ and 0.25 µM Fab’_RTX_-MORF1 + (1 h later) HSA-(MORF2)_10._

**Figure 7:**
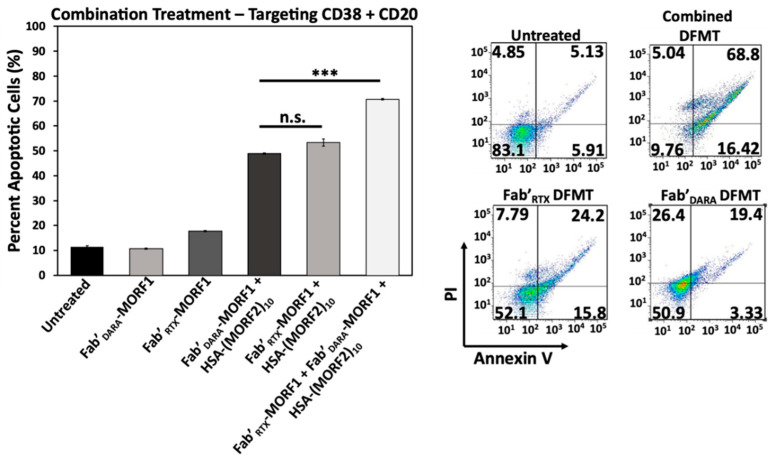
Combinational DFMT treatment of Raji cells with CD38-targeting Fab’_DARA_ DFMT coupled with CD20-targeting Fab’_RTX_ DFMT. The combined treatment was compared to individual therapies at the same MORF1 equivalence. Percent apoptotic cells were determined after DFMT treatment and analyzed with Annexin V/Propidium Iodide staining via flow cytometry. All experiments were performed in triplicate and statistically analyzed by One-Way ANOVA and Tukey test. *** *p* < 0.001, n.s. nonsignificant.

Combination treatment resulted in substantially enhanced apoptotic level (Figure 7). In addition to more effective crosslinking of receptors, probably, more signaling pathways will be involved in apoptosis initiation via two receptors when compared with one. This will be evaluated in our future research.

Notably, in relapsed non-Hodgkin lymphoma [47] and diffuse large B-cell lymphoma [48] successful treatment of patient derived xenografts was achieved by replacing anti-CD20 RTX with anti-CD38 DARA or DARA-drug conjugates. Our data combined with these results suggests that treatment of lymphoma patients with therapies involving both antibodies might be beneficial.

## 3. Methods and Materials

### 3.1. Materials

A pair of 25-mer phosphorodiamidate morpholino oligonucleotides, MORF1 and MORF2, were customized from Gene Tools (Philomath, OR, USA). In particular, MORF1 (5′-GAGTAAGCCAAGGAGAATCAATATA-3′) with 3′ primary amine modification, and its complementary MORF2 (5′-TATATTGATTCTCCTTGGCTTACTC-3′) with 3′-disulfide amide modification were used. Human serum albumin (HSA, chromatographically and fractionation purified with purity > 95%) was purchased from Innovative Research (Peary CourtNovi, MI, USA). DARA (Darzalex^®^ 20 mg/mL, Janssen Biotech (Harsham, PA, USA), ISA, OBN, and RTX were obtained from Huntsman Cancer Hospital, University of Utah. Cy3-/Cy5-NHS (*N*-hydroxysuccinimide) were purchased from Lumiprobe (Hallandale Beach, FL, USA). Tris(2-carboxyethyl) phosphine (TCEP) and heterobifunctional crosslinkers NHS-PEG_x_-maleimide (SM(PEG)_x_, x = 2, 8, and 24) were purchased from Thermo Fisher Scientific (Rockford, IL, USA). Pepsin (from porcine gastric mucosa) was from Sigma-Aldrich (St. Louis, MO, USA). Anti-Bcl-2 (100) Alexa Fluor^®^ 488 mAb and anti-Bax (2D2) Alexa Fluor^®^ 647 mAb were purchased from Santa Cruz Biotechnology (Dallas, TX, USA). Fluo-3 AM, JC-1 (5,5′,6,6′-tetrachloro-1,1′,3,3′-tetraethylbenzimidazoylcarbocyanine iodide), and carbonyl cyanide 3-chlorophenylhydrazone (CCCP) were purchased from Invitrogen (Carlsbad, CA, USA). PhiPhiLux^®^ kit was purchased from OncoImmunin (Gaithersburg, MD, USA). Lysosome tracker Green DND-26, H_2_DCFDA (2′,7′-dichlorofluorescein), Cytochrome C ELISA kit (human) and cholera toxin subunit B Alexa Fluor 555 were purchased from Thermo Fisher. All solvents were purchased from Fisher Scientific as the highest purity available.

### 3.2. Synthesis and Characterization of Nanoconjugates

#### 3.2.1. Antibody-MORF1 Nanoconjugates

Antibody-MORF1 nanoconjugates are synthesized in two steps as previously described [20]: first, a monoclonal antibody is reduced with TCEP to generate a sulfhydryl group, which is then conjugated with maleimide-modified MORF1. Here, is an example of synthesizing DARA-MORF1. DARA (225 μL, 4.5 mg) was buffer exchanged into 100 mM citrate buffer (pH 5.5) using an Amicon^®^ 4 mL ultra-centrifugal filter unit (MWCO 30,000 Da). The DARA solution was then added to 20 mM TCEP (7 mg/mL, pH rebalanced to pH 5.5), and the reaction was kept for 2 h at 37 °C water bath with gentle shaking. The reduced DARA was obtained after removal of TCEP by washing with 10 mM PBS (pH 6.5) over 6 times. In parallel, maleimide-modified MORF1 (MORF1-MAL) was prepared by reaction of MORF1-NH_2_ with 50 molar excess SM(PEG)2 (succinimidyl-[(*N*-maleimidopropionamido)-diethyleneglycol]ester). In brief, SM(PEG)2 (4.25 mg, 10 µmol) was dissolved in 50 µL DMSO, then added into 100 µL MORF1-NH_2_ solution (1.8 mg, 200 nmol) in 10 mM PBS pH 7.4. The reaction was performed for 2 h at room temperature. Then, MORF1-MAL was isolated by removal of excess SM(PEG)2 with 6 times washing using 20 mM PBS pH 6.5 buffer via Amicon^®^ 0.5 mL Ultra Centrifugal filter unit (MWCO 3000 Da). Finally, freshly reduced DARA was conjugated with MORF1-MAL in 500 µL 20 mM PBS pH 6.5 for 2.5 h at room temperature, with 1:1.5 molar ratio of [Ab]:[MORF1-MAL]. The resultant DARA-MORF1 was purified by ultracentrifugation using the Amicon^®^ 4 mL Ultra Centrifugal filter unit (MWCO 30,000 Da). The purity of conjugate DARA-MORF1 was confirmed with SEC on AKTApure with a Superdex^TM^ 200 10/300 GL column using PBS pH 7.4 as eluent. There was no detectable peak of either free MORF1 or free DARA. Furthermore, MORF1 content in the conjugate was quantified using NanoDrop (ND-1000 Spectrophotometer) at 260 nm (ε = 278,000 M^−1^cm^−1^ in 0.1 N HCl aq.), whereas the concentration of DARA was determined using a bicinchoninic acid (BCA) assay.

To prepare Cy5-labeled DARA-MORF1 (Cy5-DARA-MORF1), DARA was first labeled with Cy5-NHS. The reaction was performed with molar ratio of [Ab]:[Cy5] = 1:2 by adding Cy5-NHS solution in DMSO to DARA in 1 mL PBS pH 7.4. After 2 h reaction at room temperature, free dye was removed using a PD10 column (GE Healthcare). The collected product was then buffer exchanged using ultracentrifugation 4 times with 100 mM citric acid buffer pH 5.5. Then, procedures described above were followed.

#### 3.2.2. Fab’-MORF1 Nanoconjugates

Fab’-MORF1 nanoconjugates are synthesized in multiple steps as previously described [28]. First, a monoclonal antibody is digested into F(ab’)_2_ in the presence of pepsin, followed by reduction with TCEP to generate Fab’ with a sulfhydryl group (Fab’-SH), which is then conjugated with maleimide-modified MORF1. Here, is an example of synthesizing Fab’_DARA_-MORF1. Briefly, DARA was buffer exchanged into citrate buffer pH 4.0. Pepsin (10 *w*/*w*%) was added to DARA solution, and the reaction was kept at 37 °C. The digestion process was monitored on AKTApure till complete disappearance of DARA peak, while a new peak with lower molecular weight (at right side) showed up, which was related to F(ab’)_2_. Complete digestion occurred in 80 min. The F(ab’)_2_ was purified using ultracentrifugation with a 30,000 MWCO tube and stored at 4 °C in PBS 7.4. F(ab’)_2_ (4 mg, 4 mg/mL) was reduced with TCEP (4.6 mg, 20 mM) in 100 mM citric acid buffer pH 5.5 for 2 h at 37 °C and purified using ultracentrifugation with a 10,000 MWCO tube. The MORF1-MAL was synthesized in parallel as described above. MORF1-MAL (1.2 equiv.) was reacted with freshly reduced Fab’ (1 equiv.) for 3 h at room temperature in PBS pH 6.5 buffer. The final product was purified by ultracentrifugation using a 30,000 MWCO tube by washing 6–8 times with PBS pH 7.4 buffer. MORF1 content in Fab’_DARA_ conjugates was determined using UV-Vis absorbance at 260 nm; the concentration of antibody fragment was determined using BCA assay.

ISA-MORF1 and Fab’_ISA_-MORF1 were synthesized as described above for DARA conjugates.

#### 3.2.3. Multiple MORF2 Modified Human Serum Albumin (HSA-(MORF2)_y_)

Two steps were conducted to conjugate complementary MORF2 to HSA as previously described [29]. First, amino groups from accessible lysine residues in HSA were converted to maleimide groups by using heterobifunctional crosslinker NHS-PEG_x_-maleimide, then freshly reduced MORF2-SH was attached to HSA in multiple copies via thiol-ene reaction. In this study, SM(PEG)x (x = 2, 8, and 24) that differ in length were used in order to investigate spacer effect on MORF1-MORF2 biorecognition and induction of apoptosis. Here, is an example in which SM(PEG)_2_ was used to synthesize nanoconjugate HSA-PEG_2_-(MORF2)_10_. Briefly, HSA (5 mg, 3.9 µmol NH_2_ equiv.) was dissolved in 400 µL PBS pH 7.4 buffer. SM(PEG)2 (18.3 mg, 10 eq) in 150 µL DMSO was added into HSA solution. The reaction was kept stirring for 2 h at room temperature. The maleimide-modified HSA was then purified by ultracentrifugation using an Amicon^®^ 4 mL ultra centrifugal filter unit (MWCO 30,000 Da). The number of maleimide groups per HSA molecule was determined by a modified Ellman’s assay (for maleimide group) and BCA assay (for quantification of HSA).

In a parallel reaction, 3′-disulfide MORF2 (2.89 mg) was reduced with 3.5 mg/mL TCEP (10 mM) in 250 µL PBS pH 7.4 at 37 °C for 45 min, followed by purification via ultrafiltration using a Amicon^®^ 0.5 mL ultracentrifugal filter unit (MWCO 3000 Da). Freshly reduced MORF2-SH was then reacted with HSA-PEG_2_-MAL_x_ with molar ratio of [SH]:[MAL] = 2:1 in 500 µL PBS (pH 6.5) for 3 h at room temperature. HSA-PEG_2_-MORF2 was purified by ultracentrifugation using a 30,000 MWCO ultra centrifugal unit washing 6–8 times with PBS pH 7.4 buffer. Purity was confirmed by AKTApure. The ratio of MORF2 per HSA molecule was determined by UV-Vis spectrophotometry (ε = 252,120 M^−1^ cm^−1^ in 0.1 N HCl) and BCA assay.

For synthesis of Cy3-labeled HSA-PEG_2_-(MORF2)_y_, HSA was first reacted with Cy3-NHS with molar ratio of [HSA]:[Cy3] = 2:1. For example, a solution of Cy3-NHS (70 µg, 100 nmol) in DMSO was added to a solution of HSA (3.35 mg, 50 nmol) in 1 mL PBS pH 7.4 buffer and reacted for 2 h at room temperature. Cy3-labeled HSA was purified using a PD-10 column using PBS pH 7.4 as eluent. Then, the synthesis proceeded as described above.

#### 3.2.4. MORF1-MORF2 Hybridization

MORF1-MORF2 hybridization upon mixing Ab-MORF1 (or Fab’-MORF1) with HSA-(MORF2)_y_ was determined by the changes of optical density at 260 nm (ND-1000 spectrophotometer) that was a reflection of the hypochromic effect. For example, DARA-MORF1 (or ISA-MORF1) and HSA-(MORF2)_10_ solutions in PBS, pH 7.4 were mixed in different ratios with a constant total MORF (MORF1 + MORF2) concentration of 2.5 µM at room temperature. Then, 10 min post mixture, the optical density at 260 nm was recorded. All measurements were performed in triplicate.

In addition, MORF1-MORF2 hybridization among Ab-MORF1 (or Fab’-MORF1) and HSA-(MORF2)_10_ was also confirmed by SEC by comparison with individual conjugates, Ab-MORF1 and HSA-(MORF2)_10_.

### 3.3. Cell Culture

Human lymphoma cell lines (Daudi and Raji) and human MM cell lines (RPMI 8226 and U266) were purchased from the American Type Culture Collection (ATCC). All cells were cultured in RPMI-1640 medium supplemented with 10% fetal bovine serum (FBS), penicillin (100 units mL^−1^) and streptomycin (0.1 mg/mL^−1^) at 37 °C in a 5% CO_2_ humidified atmosphere. Human MM cell line ANBL-6 was obtained from Dr. Diane Jelinek of Mayo Clinic (Rochester, MN, USA). The cells were cultured in IMDM with 10% FBS and interleukin-6 (1 ng/mL).

### 3.4. Cell Surface CD38 Expression and Binding Assay

DARA binding experiments were conducted by incubating Daudi, RPMI 8226, Raji, ANBL-6, and U266 cells with a range of DARA (primary antibody) concentrations, and subsequently exposing them to a fluorescently labeled, anti-human secondary antibody. Specifically, 2 × 10^5^ cells were treated with DARA (1 µM, 0.5 µM, 0.25 µM, 0.1 µM or 50 nM; in PBS pH 7.4) in a 24-well plate in 400 µL media for 1 h at 4 °C. Then, the cells were washed with PBS and resuspended in PBS containing 1 *w*/*v*% BSA. An Alexa Fluor 488-labeled goat anti-human antibody (3 µL, 2 mg/mL) was added to each well and further incubated for 1 h at 4 °C. The cells were washed and resuspended in PBS and analyzed by flow cytometry. The fluorescence was normalized to an untreated control for each cell type and reported as a “fold-increase over untreated” value. All experiments were performed in triplicate.

### 3.5. MORF1-MORF2 Hybridization on Cell Surface—CD38 Crosslinking

Daudi cells (2 × 10^5^) were treated in a 24-well plate (400 µL RPMI-1640 medium per well) with Cy5-labeled DARA-MORF1 (0.5 µM MORF1) for 30 min, followed by a Cy3-labeled HSA-(MORF2)_10_ (0.5 µM [MORF2]) treatment for 1 h. Then, the cells were washed with PBS and cell nuclei were stained with Hoechst 33342 (5 µg/mL) for 5 min. The cells were washed and resuspended in PBS for imaging. Cy3 fluorescence was measured by 488 nm excitation with 530/30 nm band-pass filter. Cy5 fluorescence was measured by 633 nm excitation with 695/40 nm band-pass filter. An overlay of the respective fluorescent signals was used to visualize MORF1-MORF2 hybridization and co-localization of the nanoconjugates on the cell surfaces.

### 3.6. Apoptosis Assay

Apoptosis of DFMT-treated cells was quantified using Annexin V/Propidium Iodide staining and measured by flow cytometry. Each cell type’s (Daudi, Raji, RPMI 8226, ANBL-6, and U266) apoptotic response to DARA, DARA-based DFMT, Fab’_DARA_-based DFMT and DARA plus a secondary antibody were evaluated. Briefly, 2 × 10^5^ cells were treated in a 24-well plate in 400 µL appropriate media. Cells were first incubated with DARA-MORF1 or Fab’_DARA_-MORF1 (0.5 µM MORF1) for 1 h, followed by a PBS wash and subsequent treatment with HSA-MORF2)_10_ (0.5 µM MORF2) in fresh media for 24 h. Then, the cells were washed with Binding (HEPES saline) Buffer and stained with Annexin V/Propidium Iodide for 20 min at 4 °C. Cells were washed and resuspended in Binding Buffer for flow cytometry analysis. Untreated cells were used to gate as Annexin V/PI -/- population. Each treated sample was then compared to untreated. Values reported are the percentage of Annexin V positive cells. All treatments were performed in triplicate with appropriate controls. One-way ANOVA (α = 0.05) followed by Tukey Test analysis was used to determine significant differences in the reported data.

#### Details and Nomenclature of DFMT Apoptosis Assays

DARA DFMT: DARA-MORF1 (0.5 µM MORF1) 1 h followed by HSA-MORF2)_10_ (0.5 µM MORF2) in fresh media for 24 h; 2 × 10^5^ cells/well; 24-well plate.

Fab’_DARA_ DFMT: Fab’_DARA_-MORF1 (0.5 µM MORF1) 1 h followed by HSA-MORF2)_10_ (0.5 µM MORF2) in fresh media for 24 h; 2 × 10^5^ cells/well; 24-well plate.

ISA DFMT: ISA-MORF1 (0.5 µM MORF1) 1 h followed by HSA-MORF2)_10_ (0.5 µM MORF2) in fresh media for 24 h; 2 × 10^5^ cells/well; 24-well plate.

Fab’_ISA_ DFMT: Fab’_ISA_-MORF1 (0.5 µM MORF1) 1 h followed by HSA-MORF2)_10_ (0.5 µM MORF2) in fresh media for 24 h; 2 × 10^5^ cells/well; 24-well plate.

### 3.7. Apoptosis Inhibition

To validate the participation of calcium influx in apoptosis initiation, Daudi cells were pretreated with lipid raft inhibitor β-cyclodextrin (β-CD; inhibiting CD20 crosslinking) or Ca^2+^ chelating agent EGTA (ethyleneglycol-bis(β-aminoethyl ether)-*N*,*N*,*N′*,*N′*-tetraacetic acid) which had significantly reduced the calcium influx after DFMT treatment. Daudi cells were pretreated with either 1 mM Ca++ doped RPMI-1640 medium or 0.02% β-cyclodextrin containing RPMI-1640 medium for 1 h. The cells were washed with fresh medium and exposed to DARA-MORF1 or Fab’_DARA_-MORF1 for 1 h at 37 °C, followed by exposure to HSA-(MORF2)_10_. Cell viability was quantitated using Annexin V/PI labeling and flow cytometry.

### 3.8. Caspase 3

Caspase 3 activity was evaluated using a PhiPhiLux^®^-G_1_D_2_ kit (OncoImmunin, Gaithersburg, MD). The manufacturer’s protocol was followed, including the final stage Propidium Iodide staining to assess cell membrane integrity. The reported values are “fold-increase over control” fluorescence measurements of the PhiPhiLux indicator on treated Daudi cells over untreated cells.

### 3.9. Cytochrome C

Levels of cytochrome C were evaluated in DARA and Fab’_DARA_ DFMT-treated Daudi cells by ELISA. After treatment, Daudi cells (2 × 10^5^ cells) were washed with cold PBS and cell pellets were subsequently lysed with 100 µL cell extraction buffer (1 mM phenylmethanesulfonyl fluoride (PMSF) and 1:100 protease inhibitor cocktail) for 30 min on ice. The extract was transferred to a microcentrifuge tube and centrifuged at 13,000 rpm for 10 min at 4 °C. The amount of cytochrome C in the lysate was measured using an ELISA kit (R&D Systems) according to the manufacturer’s instructions. All samples were conducted in triplicate.

### 3.10. Mitochondrial Depolarization

JC-1 mitochondrial membrane potential sensor (Thermo Scientific) was used to evaluate the extent of mitochondrial depolarization of Daudi cells. After indicated treatments, the cells (2 × 10^5^) were washed with PBS two times and resuspended in 100 µL PBS. JC-1 (4 µM) was added to each sample and incubated at 37 °C for 30 min. For the positive control group, CCCP (0.5 µM) was added and incubated simultaneously with JC-1 for 30 min. After washing by PBS, cells were resuspended in PBS and analyzed by flow cytometry using 488 nm excitation with 530/30 nm and 585/42 nm band-pass filters, or observed under confocal microscopy. All experiments were carried out in triplicate.

### 3.11. Calcium Influx by Confocal Microscopy

Daudi cells (2 × 10^5^) were incubated with Fluo-3AM (5 µM) in 100 µL RPMI-1640 medium containing 2.5 mM Ca^2+^ for 30 min at 37 °C and compared with cells pretreated with 0.02 wt.% β-cyclodextrin or 1 mM EGTA. Following treatment, cells were washed with PBS and resuspended in RPMI-1640 medium containing 2.5 mM Ca^2+^ and observed under confocal microscopy.

### 3.12. Calcium Influx by Flow Cytometry

Raji cells (4 × 10^5^ cells/well) were counted and stained with Fluo-3 AM (5 µM) for 30 min at 37 °C. After staining, the cells were washed with PBS and resuspended in 400 µL cell culture medium containing 2.5 mM Ca^2+^ and immediately taken for flow cytometry analysis (excitation at 488 nm and emission at 530 nm). Baseline fluorescence was measured for 100 s. Then, 1 µM DARA-MORF1 or Fab’_DARA_-MORF1 was added to the sample. The fluorescence was measured continuously for another 200 s, followed by addition of HSA-(MORF2)_10_ (1 µM). Fluorescence was monitored for another 600 s or until all cells were counted. Rituximab-based DFMT calcium influx was employed as control (Figure S6).

### 3.13. Bcl-2/Bax Detection

Following treatment, levels of expression of Bcl-2 and Bax were quantified by fluorescent immunostaining. The cells were sequentially fixed by 4% paraformaldehyde for 15 min at room temperature, permeabilized by 90% methanol for 30 min on ice, and immunostained by Alexa Fluor 488 conjugated anti-Bcl-2 mAb (1:50, Santa Cruz Biotechnology) and AF647 conjugated anti-Bax mAb (1:50, Santa Cruz Biotechnology) in 1% BSA buffer for 1 h at room temperature. After washing by cold PBS twice, the fluorescence was quantified by flow analysis. All experiments were carried out in triplicate.

### 3.14. Lysosomal Enlargement

Type II antibody-induced cell death involves lysosomal enlargement/breakage. Lysosome activity was tested by LysoTracker Green staining followed by flow cytometry. Raji cells (2 × 10^5^) were exposed to naked antibody or antibody-MORF1 conjugate for 1 h at 37 °C, followed by exposure to HSA-(MORF2)_10_. After 24 h treatment, cells were stained with LysoTracker Green DND-26 (200 nM) for 20 min at 37 °C. Fluorescence was quantified using flow cytometry. Each sample was prepared in triplicate.

### 3.15. Reactive Oxygen Species Production

Quantification of reactive oxygen production was performed on Raji cells (2 × 10^5^) by oxidation of 2′,7′-dichlorodihydrofluorescein diacetate (H_2_DCFDA). Cells (2 × 10^5^ cell/well, 24-well plate) were treated with antibody (DAR, ISA, or OBN) (0.5 µM) or antibody (DARA, ISA, or OBN)-MORF1 conjugate followed by HSA-(MORF2)_10_ (MORF1 = MORF2 = 0.5 μM). After 24 h treatment, cells were incubated with H_2_DCFDA (5 μM) for 30 min at 37 °C. Cells were washed with PBS and analyzed with flow cytometry. Each sample was prepared in triplicate.

### 3.16. Translocation of CD38 into Lipid Rafts

The motility and translocation of CD38 receptors under the influence of DFMT was evaluated by cholera toxin B staining and observed under confocal microscopy. Briefly, Raji cells (2 × 10^5^ cells/well) were loaded into a 24-well plate. The cells were treated with either 0.5 μM antibody alone or 0.5 μM Cy5-labeled antibody-MORF1 for 1 h at 37 °C followed by PBS wash and 0.5 μM HSA-(MORF2)_10_ exposure for 2 h at 37 °C. Then, the cells were washed to remove unbound antibodies, and stained with Alexa Fluor-555 conjugated cholera toxin B subunit (10 μg/mL) for 1 h at 4 °C. The samples were immediately imaged by confocal microscopy.

### 3.17. Statistical Analysis

All statistical analysis was performed on Microsoft Excel. No samples were left out of any analysis calculations. Sample groups were compared using one-way ANOVA followed by Tukey test. *p* < 0.05 was considered statistically significant. All experiments were performed with at least *n = 3* samples per group. Percent apoptotic cells was calculated by summation of quadrants one, two and four in the Annexin V/PI gated flow cytometry runs. Magnitude of fluorescence in immunostaining procedures was quantified by flow cytometry’s geometric mean average function. The geometric mean average was either normalized to untreated control cells or presented as fold increase over untreated, as specified above.

## 4. Conclusions

The experiments presented herein illustrate the versatility of the DFMT system and demonstrate how it can be applied towards the treatment of several B cell malignancies including multiple myeloma, lymphoma and leukemia. Four novel antibody nanoconjugates as bispecific engagers (Fab’_DARA_-MORF1, Fab’_ISA_-MORF1, DARA-MORF1, and ISA-MORF1) and adaptations to the HSA-based multivalent crosslinking effector molecule (HSA-PEG_x_-(MORF2)_y_) were synthesized. Biorecognition of complementary engagers and effectors at cell surface mediated by MORF1/MORF2 hybridization resulted in crosslinking of CD38 receptors and apoptosis initiation in CD38+ cells: Daudi, Raji RPMI 8226 and ANBL-6. The level of apoptosis induction, Daudi > RPMI 8226 > Raji > ANBL-6 >> U266, correlated with CD38 expression. Additionally, insertion of a flexible PEG_24_ (95.2 Å) spacer into the effector conjugate HSA-PEG_24_-(MORF)_13_ significantly increased apoptosis of Raji cells when compared to effectors containing PEG_8_ (39.2 Å) or PEG_2_ (17.6 Å) moieties.

Beyond the synthesis and apoptosis efficacy studies with the new conjugates, a thorough investigation into the mechanisms of action of DARA, DARA DFMT, and Fab’_DARA_ DFMT on CD38+ cell lines were conducted. Preincubation of cells with β-cyclodextrin (to extract cholesterol) or EGTA (to complex extracellular Ca++) decreased levels of apoptosis. DARA-based and Fab’_DARA_-based DMFT induced, following crosslinking of CD38 receptors, apoptosis via the mitochondrial signaling pathway as indicated by enhanced Bax/Bcl-2 expression ratio, ROS generation, cytochrome C release, and caspase 3 activation.

A comparison of ISA and ISA-based DFMT to DARA and a known Type II antibody, OBN was conducted. ISA induced apoptosis in Daudi and Raji cells. ISA DFMT did not enhance apoptosis when compared to ISA; however, crosslinking of the CD38-Fab’_ISA_-MORF1 complex with HSA-(MORF2)_10_ resulted in enhanced apoptotic levels. Additionally, Fab’_ISA_-MORF1 induced apoptosis in Daudi and Raji cells on its own. Comparison with DFMT based on anti-CD20 Type II antibody OBN revealed that both DARA and ISA did not exhibit features related to Type II apoptotic mechanisms (lysosomal enlargement, homotypic cell adhesion).

Finally, simultaneous crosslinking of CD38 and CD20 receptors on Raji cells increases the level of apoptosis when compared to crosslinking of individual receptors. This finding suggests a therapeutic potential of lymphoma treatment with a mixture of antibodies.

## Data Availability

All data generated or analyzed during this study are included in the article.

## Supplementary Materials

The following are available online. Figure S1. Size exclusion chromatography (SEC) of DARA-MORF1 and HSA-(MORF2)_10_ hybridization. Figure S2. UV-Vis spectroscopy to determine DARA-MORF1 and HSA-(MORF2)_10_ hybridization. Figure S3. SEC of Fab’_ISA_-MORF1 and its intermediates. Figure S4. SEC of ISA-MORF1 and HSA-(MORF2)_10_ hybridization. Figure S5. UV-Vis spectroscopy to determine ISA-MORF1 and HSA-(MORF2)_10_ hybridization. Figure S6. Flow cytometry cell population shifts for DARA DFMT experiments. Figure S7. Flow cytometry cell population shifts for Fab’_DARA_ DFMT experiments. Figure S8. DARA DFMT on ANBL-6 cells. Figure S9. Flow cytometry readout of Rituximab DFMT to monitor calcium influx. Figure S10. Confocal microscopy of Fab’_DARA_ DFMT apoptosis inhibition by β-CD and EGTA. Figure S11. Bax/Bcl-2 expression of DFMT-treated Daudi cells. Figure S12. Cytochrome C calibration curve from ELISA assay. Figure S13. Flow cytometry of Bax and Bcl-2 expression in Daudi cells. Figure S14. Flow cytometry of caspase 3 population gating. Figure S15. Flow cytometry of caspase 3 post-measurement propidium iodide staining. Figure S16. Confocal microscopy of assessment of lipid raft redistribution using Cy5-labeled antibodies. Figure S17. Additional confocal microscopy of lipid raft redistribution induced by DFMT systems. Figure S18. ISA DFMT & Fab’_ISA_ DFMT apoptosis assessment on Raji cells.

## Sample Availability

Samples of the compounds are not available from the authors.

## References

[1] Im A., Pavletic S.Z. (2017). Immunotherapy in hematologic malignancies: Past, present, and future. J. Hematol. Oncol..

[2] Meyer S., Evers M., Jansen J.H.M., Buijs J., Broek B., Reitsma S.E., Moerer P., Amini M., Kretschmer A., Ten Broeke T. (2018). New insights in Type I and Type II CD20 antibody mechanisms of action with a panel of novel CD20 antibodies. Br. J. Haematol..

[3] Moody P.R., Sayers E.J., Magnusson J.P., Alexander C., Borri P., Watson P., Jones A.T. (2015). Receptor crosslinking: A general method to trigger internalization and lysosomal targeting of therapeutic receptor: Ligand complexes. Mol. Ther..

[4] Radford D.C., Yang J., Doan M., Li L., Dixon A.S., Owen S.C., Kopeček J. (2020). Multivalent HER2-binding polymer conjugates facilitate rapid endocytosis and enhance intracellular drug delivery. J. Control. Release.

[5] Li L., Li Y., Yang J., Radford D.C., Wang J., Janát-Amsbury M., Kopeček J., Yang J. (2020). Inhibition of immunosuppresive tumors by polymer-assisted inductions of immunogenic cell death and multivalent PD-L1 crosslinking. Adv. Funct. Mater..

[6] Overdijk M.B., Jansen J.H., Nederland M., Lammerts van Bueren J.J., Groen R.W., Parren P.W., Leusen J.H., Boross P. (2016). The therapeutic CD38 monoclonal antibody daratumumab induces programmed cell death via Fcγ receptor-mediated crosslinking. J. Immunol..

[7] van de Donk N.W.C.J., Usmani S.Z. (2018). CD38 antibodies in multiple myeloma: Mechanism of action and modes of resistance. Front. Immunol..

[8] Sanchez L., Wang Y., Siegel D.S., Wang M.L. (2016). Daratumumab: A first-in-class CD38 monoclonal antibody for the treatment of multiple myeloma. J. Hematol. Oncol..

[9] Richter J., Sanchez L., Thibaud S. (2020). Therapeutical potential of isatuximab in the treatment of multiple myeloma. Semin. Oncol..

[10] van de Donk N.W.C.J., Richardson P.G., Malavasi F. (2018). CD38 antibodies in multiple myeloma: Back to the future. Blood.

[11] Wu K., Liu J., Johnson R.N., Yang J., Kopeček J. (2010). Drug-free macromolecular therapeutics: Induction of apoptosis by coiled-coil mediated crosslinking of antigens on cell surface. Angew. Chem. Int. Ed..

[12] Yang J., Li L., Kopeček J. (2019). Biorecognition: A key to drug-free macromolecular therapeutics. Biomaterials.

[13] Rütter N.M., Milošević A. (2021). David, Say no to drugs: Bioactive macromolecular therapeutics without conventional drugs. J. Control. Release.

[14] Chu T.-W., Zhang R., Yang J., Chao M.P., Shami P.J., Kopeček J. (2015). A Two-step pretargeted nanotherapy for CD20 crosslinking may achieve superior anti-lymphoma efficacy to Rituximab. Theranostics.

[15] Hartley J.M., Chu T.-W., Peterson E.M., Zhang R., Yang J., Harris J., Kopeček J. (2015). Super-resolution imaging and quantitative analysis of membrane protein/lipid raft clustering mediated by cell surface self-assembly of hybrid nanoconjugates. ChemBioChem.

[16] Wu K., Yang J., Liu J., Kopeček J. (2012). Coiled-coil based drug-free macromolecular therapeutics: In vivo efficacy. J. Control. Release.

[17] Li L., Yang J., Wang J., Kopeček J. (2018). Amplification of CD20 crosslinking in Rituximab resistant B-lymphoma cells enhances apoptosis induction by drug-free macromolecular therapeutics. ACS Nano.

[18] Wang J., Li L., Yang J., Clair P.M., Glenn M., Stephens D.M., Radford D.C., Kosak K.M., Deininger M.W., Shami P.J. (2019). Drug-free macromolecular therapeutics induce apoptosis in cells isolated from patients with B cell malignancies with enhanced apoptosis induction by pretreatment with gemcitabine. Nanomedicine NBM.

[19] Li L., Yang J., Wang J., Kopeček J. (2018). Drug-free macromolecular therapeutics induce apoptosis via calcium influx and mitochondrial signaling pathway. Macromol. Biosci..

[20] Bhatnagar V., Gormley N.J., Luo L., Shen Y.L., Sridhara R., Subramaniam S., Shen G., Ma L., Shord S., Glodberg K.B. (2017). FDA approval summary: Daratumumab for treatment of multiple myeloma after one prior therapy. Oncologist.

[21] Gabellier L., Carton G. (2016). Obinutuzumab for relapsed or refractory indolent non-Hodgkin’s lymphomas. Ther. Adv. Hematol..

[22] Jiang H., Acharya C., An G., Zhong M., Feng X., Wang L., Dasilva N., Song Z., Yang G., Adrian F. (2016). SAR650984 directly induces multiple myeloma cell death via lysosomal-associated and apoptotic pathways, which is further enhanced by pomalidomide. Leukemia.

[23] Wang S., Liu S., Zhang Y., He J., Coy D. (2020). Human serum albumin (HSA) and its applications as a drug delivery vehicle. Health Sci. J..

[24] Benizri S., Gissot A., Martin A., Vialet B., Grinstaff M.W., Barthélémy P. (2019). Bioconjugated oligonucleotides: Recent developments and therapeutic applications. Bioconjug. Chem..

[25] Kverka M., Hartley J.M., Chu T.-W., Yang J., Heidchen R., Kopeček J. (2014). Immunogenicity of coiled-coil based drug-free macromolecular therapeutics. Biomaterials.

[26] Seyfizadeh N., Seyfizadeh N., Hasenkamp J., Huerta-Yepez S. (2016). A molecular perspective on rituximab: A monoclonal antibody for B cell non-Hodgkin lymphoma and other affections. Crit. Rev. Oncol. Hemat..

[27] Li L., Wang J., Li Y., Radford D.C., Yang J., Kopeček J. (2019). Broadening and enhancing functions of antibodies by self-assembling multimerization at cell surface. ACS Nano.

[28] Zhang L., Fang Y., Yang J., Kopeček J. (2017). Drug-free macromolecular therapeutics: Impact of structure on induction of apoptosis in Raji B cells. J. Control. Release.

[29] Li L., Yang J., Soodvilai S., Wang J., Opanasopit P., Kopeček J. (2019). Drug-free albumin-triggered sensitization of cancer cells to anticancer drugs. J. Control. Release.

[30] Funaro A., Reiniš M., Trubiani O., Santi S., Di Primio R., Malavasi F. (1998). CD38 functions are regulated through internalization step. J. Immunol..

[31] Ghose J., Viola D., Terrazas C., Caserta E., Troadec E., Khalife J., Gulsen Gunes E., Sanchez J., McDonald T., Marcucci G. (2018). Daratumumab induces CD38 internalization and impairs myeloma cell adhesion. Oncoimmunology.

[32] Chu T.-W., Kopeček J. (2015). Drug-free macromolecular therapeutics – A new paradigm in polymeric nanomedicines. Biomater. Sci..

[33] Goodwin D.A., Meares C.F. (2001). Advances in pretargeting biotechnology. Biotechnol. Adv..

[34] Sharkey R.M., Karacay H., Cardillo T.M., Chang C.H., McBride W.J., Rossi E.A., Horak I.D., Goldenberg D.M. (2005). Improving the delivery of radionuclides for imaging and therapy of cancer using pretargeting methods. Clin. Cancer Res..

[35] Liu G., Dou S., Ruscowski M., Hnatowich D.J. (2008). An experimental and theoretical evaluation of the influence of pretargeting antibody on the tumor accumulation of effector. Mol. Cancer Res..

[36] Niwa T., Kasuya Y., Suzuki Y., Ichikawa K., Yoshida H., Kurimoto A., Tanaka K., Morita K. (2018). Novel immunoliposome technology for enhancing the activity of the agonistic antibody against the tumor necrosis factor receptor superfamily. Mol. Pharm..

[37] Semac I., Palomba C., Kulangara K., Klages N., van Echten-Deckert G., Borisch B., Hoessli D.C. (2003). Anti-CD20 therapeutic antibody rituximab modifies the functional organization of rafts/microdomains of B lymphoma cells. Cancer Res..

[38] Li Y.C., Park M.J., Ye S.-K., Kim C.-W., Kim Y.-N. (2006). Elevated levels of cholesterol-rich lipid rafts in cancer cells are correlated with apoptosis sensitivity induced by cholesterol-depleting agents. Am. J. Pathol..

[39] Janas E., Priest R., Wilde J.I., White J.H., Malhorta R. (2005). Rituxan (anti-CD20 antibody)-induced translocation of CD20 into lipid rafts is crucial for calcium influx and apoptosis. Clin. Exp. Immunol..

[40] Kaufmann S.H., Hengartner M.O. (2001). Programmed cell death: Alive and well in the next millenium. Trends Cell Biol..

[41] Saxena A., Viswanathan S., Moshynska O., Tandon P., Sankaran K., Sheridan D.P. (2004). Mcl-1 and Bcl-2/Bax ratio are associated with treatment response but not with Rai stage in B-cell chronic lymphocytic leukemia. Am. J. Hematol..

[42] Fletcher A.I., Meusburger S., Hawkins C.J., Riglar D.T., Lee E.F., Fairlie W.D., Huang D.C.S., Adams J.M. (2008). Apoptosis is triggered when prosurvival Bcl-2 proteins cannot restrain Bax. Proc. Natl. Acad. Sci. USA.

[43] Kim R., Emi M., Tanabe K. (2006). Role of mitochindria as the gradens of cell death. Cancer Chemother. Pharmacol..

[44] Alduaij W., Ivanov A., Honeychurch J., Cheadle E., Potluri S., Lim S.H., Shimada K., Chan C.H., Tutt A., Beers S.A. (2010). Novel type II anti-CD20 monoclonal antibody (GA101) evokes homotypic adhesion and actin-dependent, lysosome-mediated cell death in B-cell malignancies. Blood.

[45] Deckert J., Wetzel M.-C., Bartle L.M., Skaletskaya A., Goldmacher V.S., Valée F., Zhou-Liu Q., Ferrari P., Pouznieux S., Lahoute C. (2014). SAR650984, A novel humanized CD38-targeting antibody, demonstrates potent antitumor activity in models of multiple myeloma and other CD38+ hematologic malignancies. Clin. Cancer Res..

[46] Moreno L., Perez C., Zabaleta A., Manrique I., Alignani D., Ajona D., Blanco D., Lasa M., Maiso P., Rodriguez I. (2019). The mechanism of action of the anti-CD38 monoclonal antibody isatuximab in multiple myeloma. Clin. Cancer Res..

[47] Vočková P., Svatoň M., Karolová J., Pokorná E., Vokurka M., Klener P. (2020). Anti-CD38 therapy with daratumumab for relapsed/refractory CD20-negative diffuse large B-cell lymphoma. Folia Biol. (Praha).

[48] Lidický O., Klener P., Machová D., Vočková P., Pokorná E., Helman K., Mavis C., Janoušková O., Etrych T. (2020). Overcoming resistence to rituximab in relapsed non-Hodgkin lymphomas by antibody-polymer drug conjugates actively targeted by anti-CD38 daratumumab. J. Control. Release.

