# Supplementary material for: Crosslinking of CD38 Receptors Triggers Apoptosis of Malignant B Cells
Source: Molecules. 2021 Jul 31;26(15):4658. doi: 10.3390/molecules26154658 (PMC8348492; doi:10.3390/molecules26154658)
Supplement: Supplementary file 1 [file molecules-26-04658-s001.zip › molecules-1269061-supplementary.pdf]

Supporting Information

Crosslinking of CD38 receptors triggers apoptosis of malignant B cells

M. Tommy Gambles[1,2], Jiahui Li[1,2], Jiawei Wang[1,2], Douglas Sborov[3] Jiyuan Yang[1,2,*], Jindřich Kopeček[1,2,4*]

[1] Center for Controlled Chemical Delivery, University of Utah, Salt Lake City, UT 84112, USA
[2] Department of Pharmaceutics and Pharmaceutical Chemistry, University of Utah, Salt Lake City, UT 84112, USA
[3] Huntsman Cancer Institute, University of Utah, Salt Lake City, UT 84112, USA
[4] Department of Biomedical Engineering, University of Utah, Salt Lake City, Utah 84112, USA
* Correspondence: jindrich.kopecek@utah.edu; jiyuan.yang@utah.edu

Table of Contents

Figure S1. Size exclusion chromatography (SEC) of DARA-MORF1 and HSA-(MORF2)$_{10}$ hybridization.

Figure S2. UV-Vis spectroscopy to determine DARA-MORF1 and HSA-(MORF2)$_{10}$ hybridization.

Figure S3. SEC of Fab'$_{ISA}$-MORF1 and its intermediates.

Figure S4. SEC of ISA-MORF1 and HSA-(MORF2)$_{10}$ hybridization.

Figure S5. UV-Vis spectroscopy to determine ISA-MORF1 and HSA-(MORF2)$_{10}$ hybridization.

Figure S6. Flow cytometry cell population shifts for DARA DFMT experiments.

Figure S7. Flow cytometry cell population shifts for Fab'$_{DARA}$ DFMT experiments.

Figure S8. DARA DFMT on ANBL-6 cells.

Figure S9. Flow cytometry readout of Rituximab DFMT to monitor calcium influx.

Figure S10. Confocal microscopy of Fab'$_{DARA}$ DFMT apoptosis inhibition by β-CD and EGTA.

Figure S11. Bax/Bcl-2 expression of DFMT-treated Daudi cells.

Figure S12. Cytochrome C calibration curve from ELISA assay.

Figure S13. Flow cytometry of Bax and Bcl-2 expression in Daudi cells.

Figure S14. Flow cytometry of caspase 3 population gating.

Figure S15. Flow cytometry of caspase 3 post-measurement propidium iodide staining.

Figure S16. Confocal microscopy of assessment of lipid raft redistribution using Cy5-labeled antibodies.

Figure S17. Additional confocal microscopy of lipid raft redistribution induced by DFMT systems.

Figure S18. ISA DFMT & Fab'$_{ISA}$ DFMT apoptosis assessment on Raji cells.

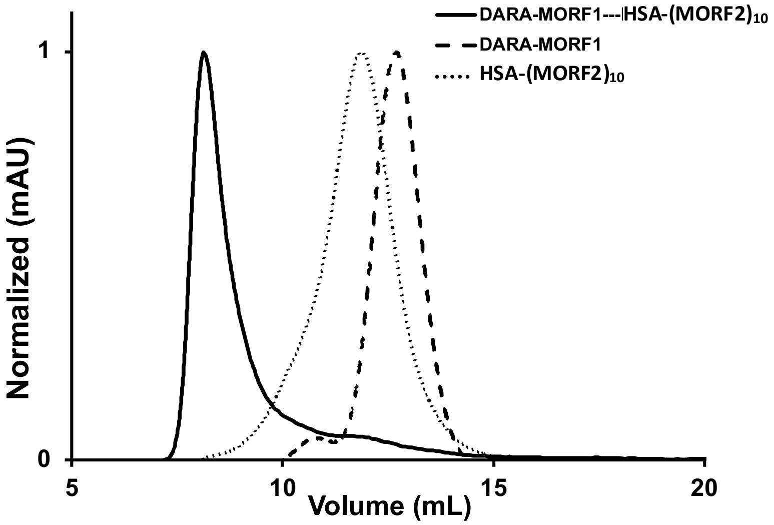

Figure S1. DARA-MORF1 and HSA-$(MORF2)_{10}$ hybridization determined by SEC with a Superdex 200 10/300 GL column, PBS (pH 7.4) as eluant at 0.4 mL/min flow rate. A 1:1 MORF1:MORF2 molar equivalent solution of DARA-MORF1 and HSA-$(MORF2)_{10}$ was premixed in PBS and allowed to hybridize for 20 min. Hybridized nanoconjugate (solid) was compared to DARA-MORF1 (dashed) and HSA-$(MORF2)_{10}$ (dotted).

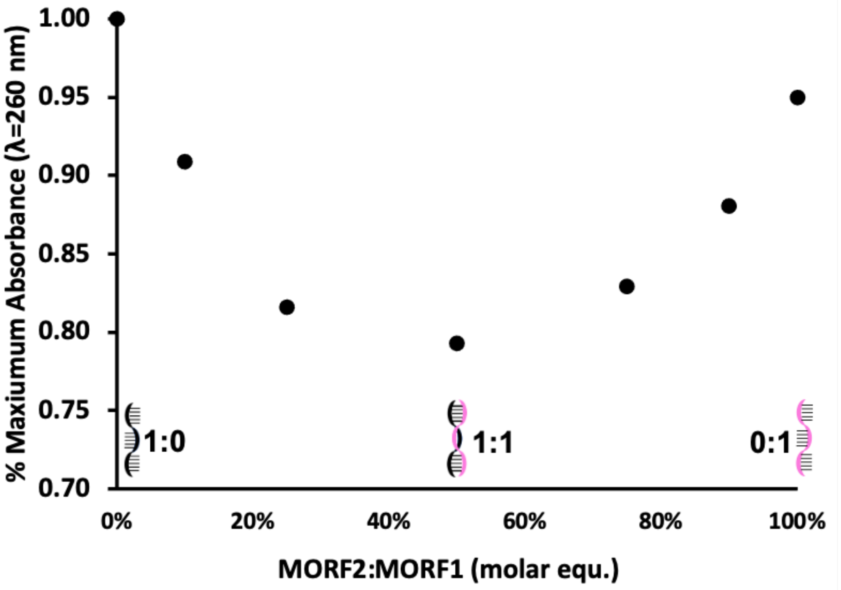

Figure S2. UV-Vis spectrophotometry observations of morpholino hybridization between DARA-MORF1 and HSA-$(MORF2)_{10}$ nanoconjugates. Absorbance at λ = 260 nm was monitored for base pair complementation with varying molar equivalences of MORF1:MORF2 in a 0.1 M HCl solution ε_{MORF1} = 278,000 M^{-1} cm^{-1}; ε_{MORF2} = 252,120 M^{-1} cm^{-1}. Hybridization is indicated by a decrease in absorbance at this wavelength.

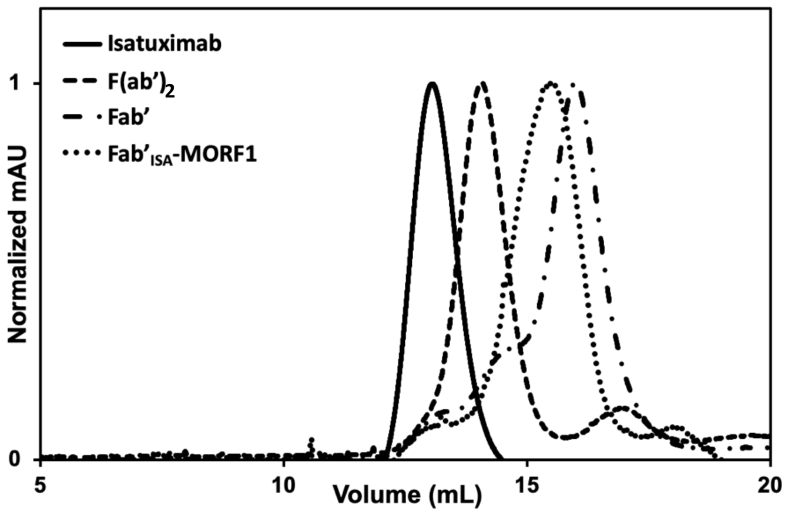

Figure S3. SEC of Fab'$_{ISA}$-MORF1 and its intermediates with a Superdex 200 10/300 GL column, PBS (pH 7.4) as eluant at 0.4 mL/min flow rate.

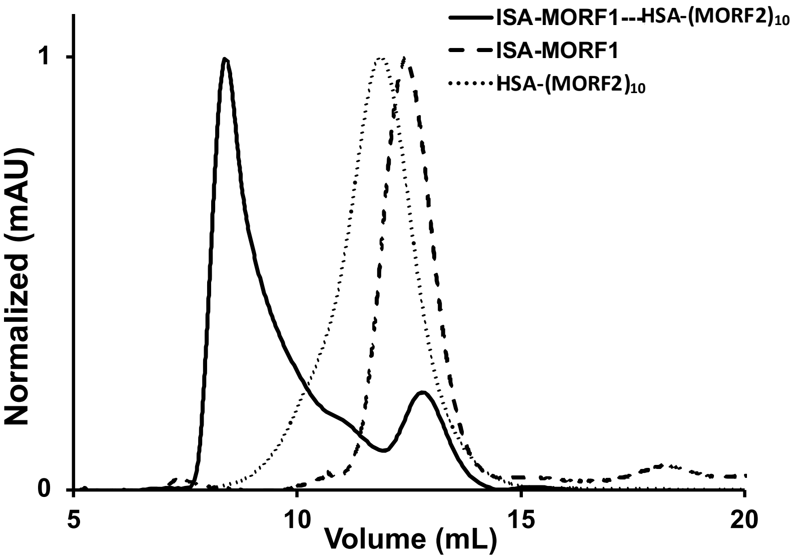

Figure S4. ISA-MORF1 and HSA-$(MORF2)_{10}$ hybridization determined by SEC with a Superdex 200 10/300 GL column, PBS (pH 7.4) as eluant at 0.4 mL/min flow rate. A 1:1 MORF1:MORF2 molar equivalent solution of ISA-MORF1 and HSA-$(MORF2)_{10}$ was premixed in PBS and allowed to hybridize for 20 min. Hybridized nanoconjugate (solid) was compared to ISA-MORF1 (dashed) and HSA-$(MORF2)_{10}$ (dotted).

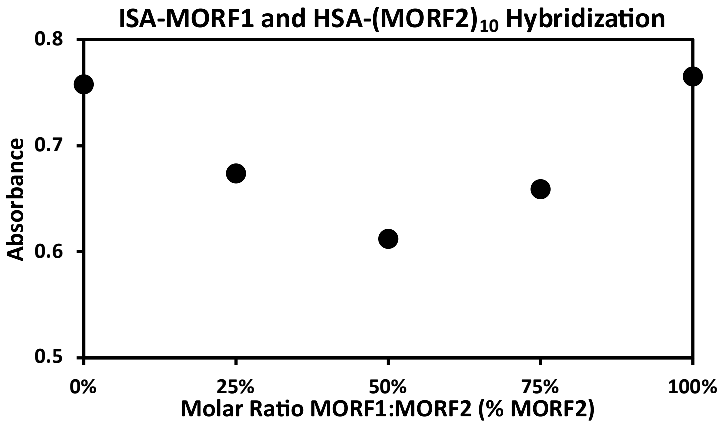

Figure S5. UV-Vis spectroscopy observations of morpholino hybridization between ISA- MORF1 and HSA-$(MORF2)_{10}$ nanoconjugates. Absorbance at λ = 260 nm was monitored for base pair complementation with varying molar equivalences of MORF1:MORF2 in a 0.1 M HCl solution ε_{MORF1} = 278,000 M^{-1} cm^{-1}; ε_{MORF2} = 252,120 M^{-1} cm^{-1}. Hybridization is indicated by a decrease in absorbance at this wavelength.

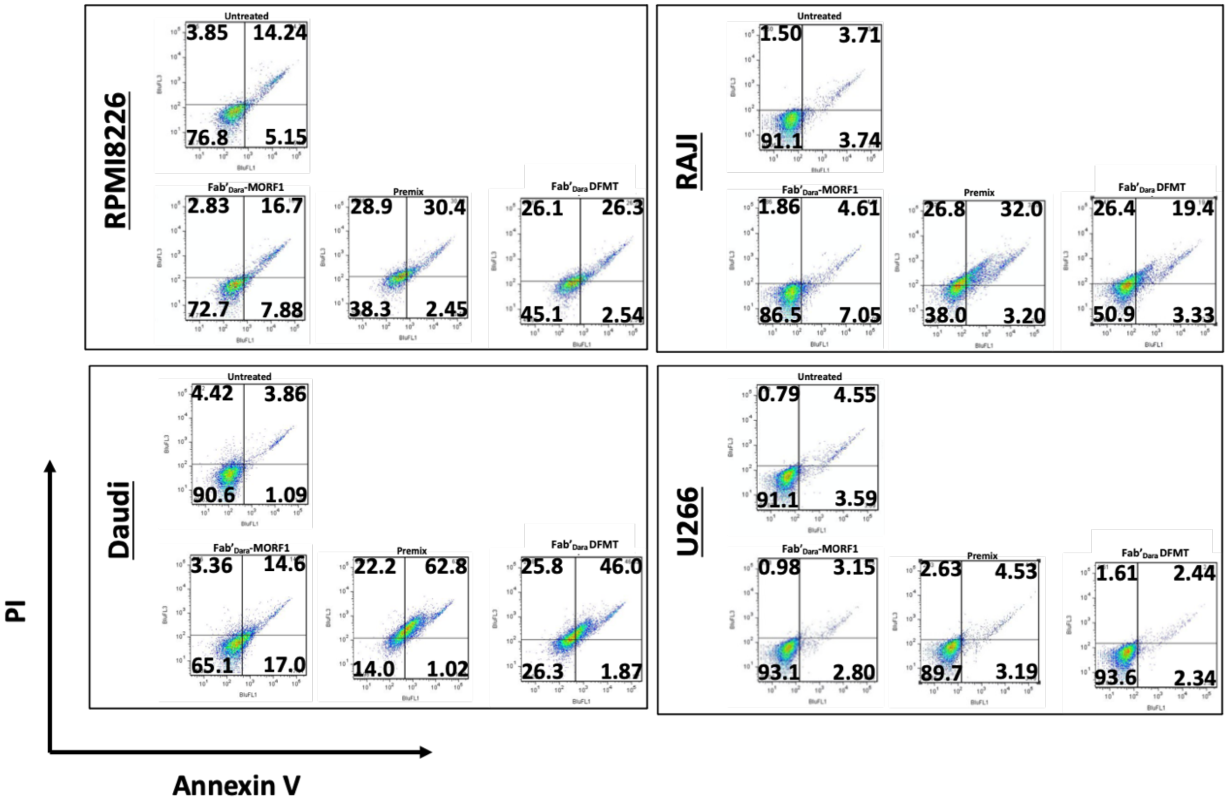

Figure S6. Flow cytometry cell populations of Fab'$_{DARA}$ DFMT on four B cells: Raji, Daudi, RPMI 8226 and U 266. Experiments were performed in triplicate (only one of each sample shown for brevity). Test samples include: Untreated, Fab'$_{DARA}$-MORF1 alone, HSA-$(MORF2)_{10}$ alone, Premix, and Fab'$_{DARA}$-MORF1 DFMT. Each DFMT treatment was performed with 0.5 µM MORF1 and 0.5 µM MORF2 concentrations. The Premix sample included a premixed solution of Fab'$_{DARA}$-MORF1 and HSA-$(MORF2)_{10}$ in PBS allowed to pre-hybridize for 20 min.

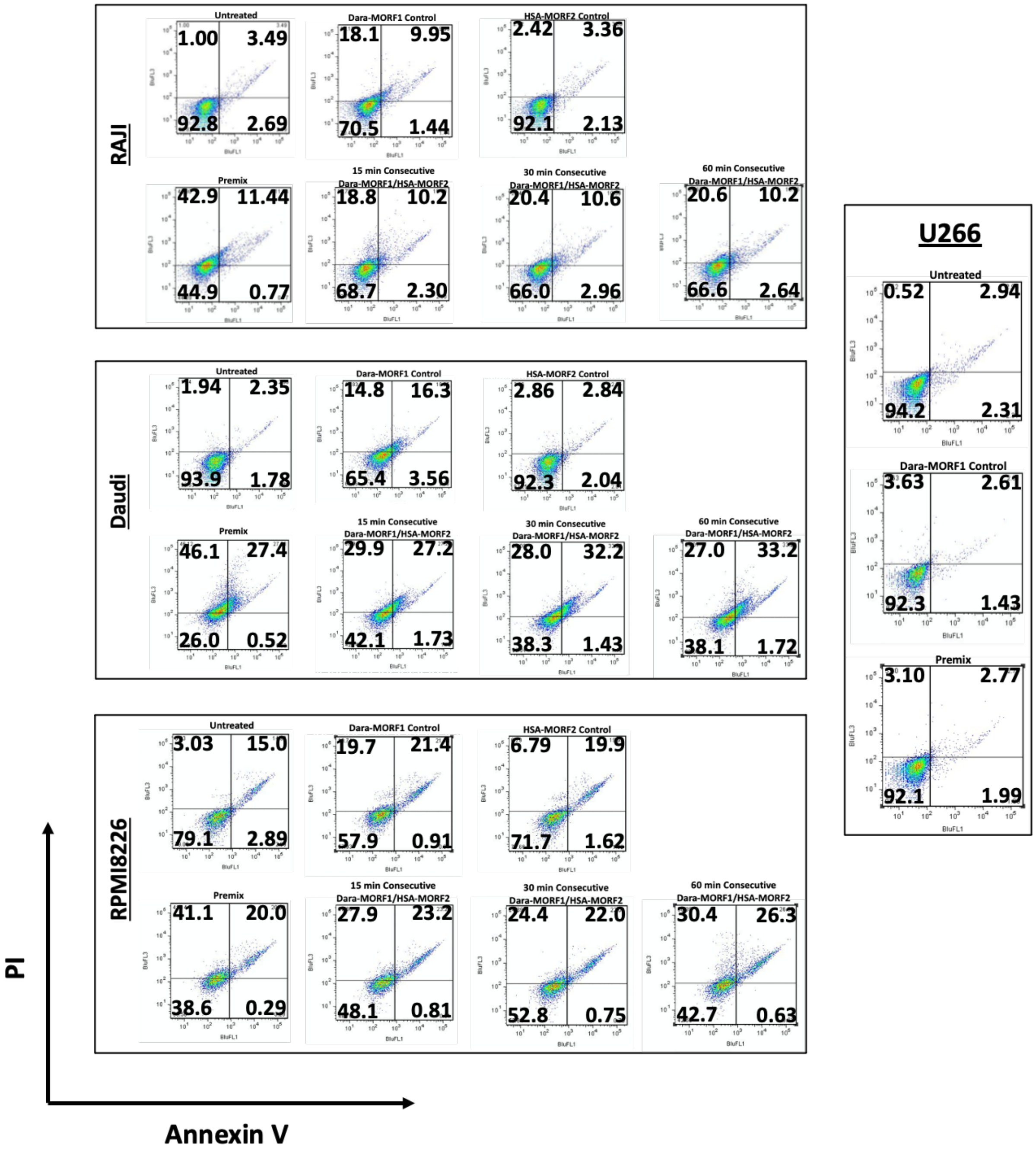

Figure S7. Flow cytometry cell populations of DARA DFMT on the four B cell types: Raji, Daudi, RPMI 8226 and U 266. Experiments were performed in triplicate (only one of each sample shown for brevity). Test samples include: Untreated, DARA-MORF1 alone, HSA-$(MORF2)_{10}$ alone, Premix; and varying DARA-MORF1 treatment times of 15 min, 30 min and 60 min followed by PBS wash and HSA-$(MORF2)_{10}$ treatment for 24 h. Each DFMT treatment was performed with 0.5 µM MORF1 and 0.5 µM MORF2 concentrations. The *Premix* sample included a premixed solution of DARA-MORF1 and HSA-$(MORF2)_{10}$ in PBS allowed to pre-hybridize for 20 min. Corresponds with Figure 2D of the main text.

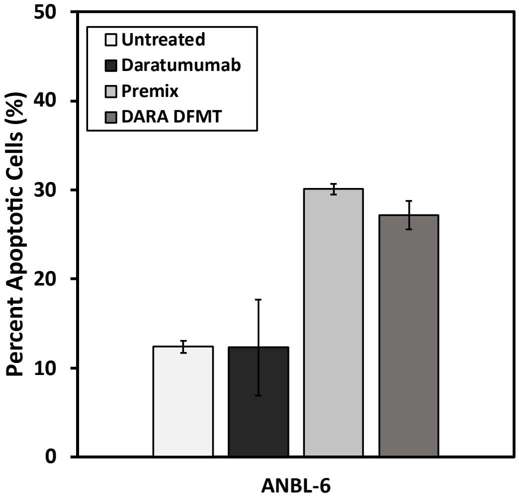

Figure S8. DARA DFMT on ANBL-6 cells (multiple myeloma). DFMT showed significant response over naked antibody, but therapeutic efficacy was limited due to low CD38 expression (determined by DARA binding studies – Figure 2A). Future work will investigate methods to increase CD38 expression to improve therapeutic response on low or moderately CD38-expressing cell types.

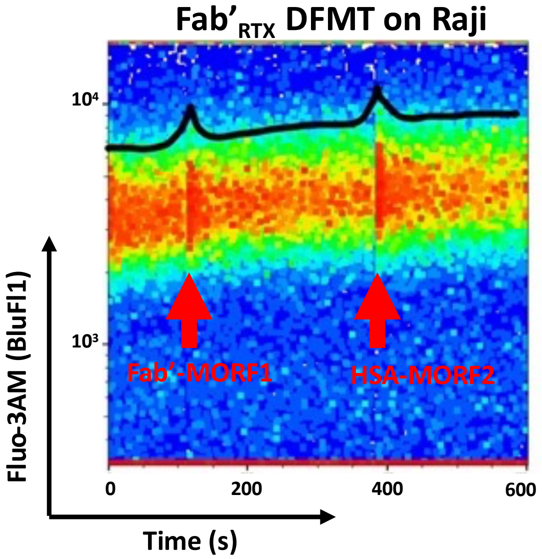

Figure S9. Calcium influx monitoring via continuous flow cytometry using Fab'$_{RTX}$ DFMT. We have previously shown calcium influx with RTX DFMT on Raji cells. Experiment was repeated herein as positive control of the calcium influx experiments. The first peak indicates surge of fluorescence by the calcium indicator at the time Fab'$_{RTX}$-MORF1 was added to the cell suspension. The second peak indicates a surge of fluorescence by the calcium indicator induced by the addition of HSA-(MORF2)$_{10}$ into the cell suspension. Corresponds with Figure 3B of the main text.

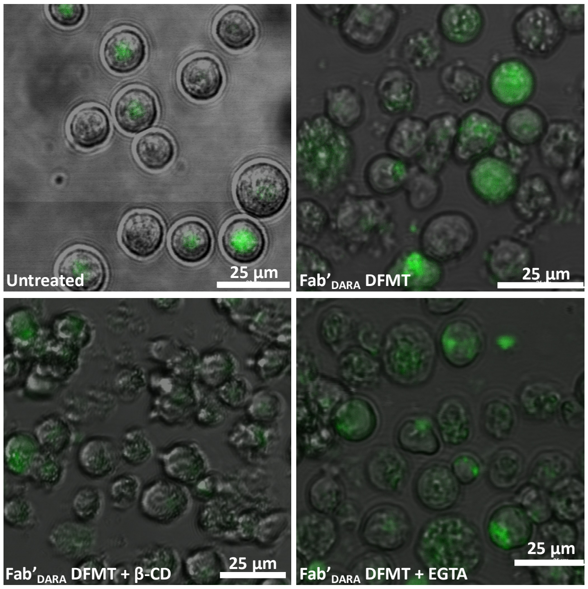

Figure S10. Fab'$_{DARA}$ DFMT inhibition with β-CD and EGTA examined by confocal fluorescence microscopy using Fluo-3AM fluorescent calcium indicator. DFMT was performed on Daudi cells with and without inhibitors compared to untreated cells in the same method describe for DARA DFMT in Figure 3C.

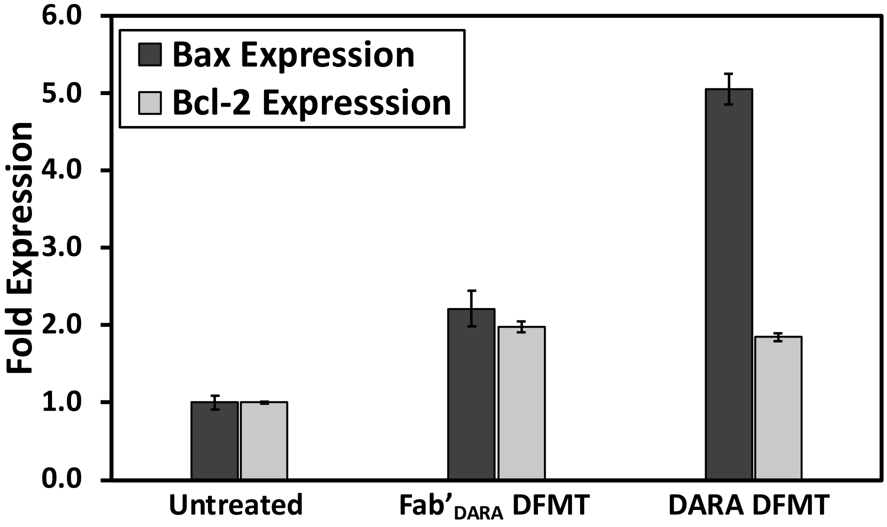

Figure S11. Bax and Bcl-2 protein expression data from Dara-based DFMT treated Daudi cells compared to untreated. Expression was measured using immunostaining and flow cytometry to compare fluorescence of treated cells to untreated cells. Higher ratio of Bax to Bcl-2 indicates cells are in apoptotic state. Corresponds with Figure 4B of the main text.

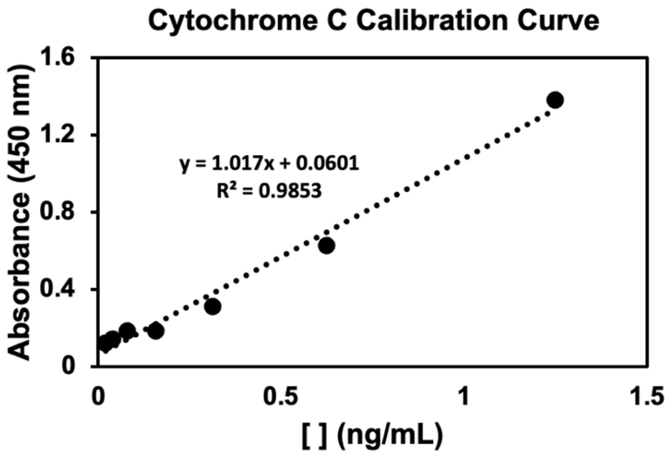

Figure S12. Cytochrome C calibration curve generated with standards from ELISA kit. Corresponds with cytochrome C data in Figure 4D.

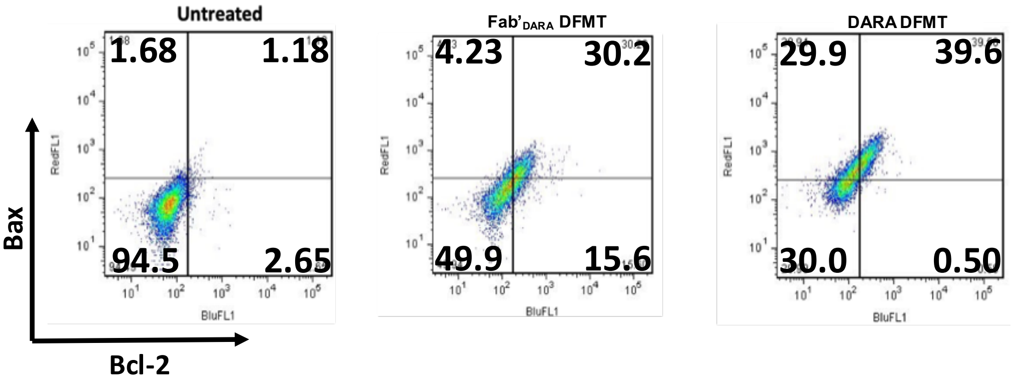

Figure S13. Flow cytometry cell population shifts of Daudi cells treated with DARA and Fab'$_{DARA}$ DFMT. Bax expression was assayed using AF647 conjugated anti-Bax mAb. Bcl-2 expression was assayed using Alexa Fluor 488 conjugated anti-Bcl-2 mAb. Experiment was performed in triplicate. Corresponds with Figure 4B of the main text.

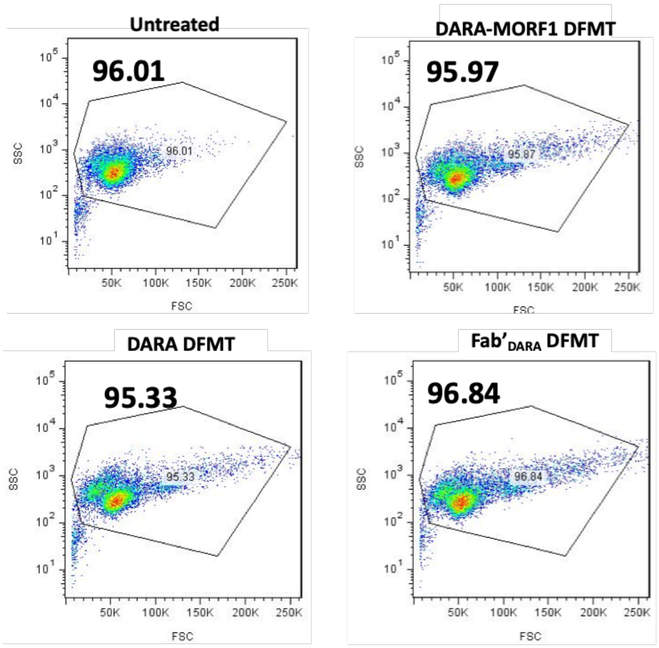

Figure S14. Cell population gating on flow cytometry of untreated cells versus DFMT-treated Daudi cells for the caspase 3 experiment. Signature apoptotic population appearance with tail of aggregates was gated while excluding ruptured/dead population at sub-20K FSC. Experiment was performed in triplicate and corresponds with the caspase 3 bar graph Figure 4C in the main text.

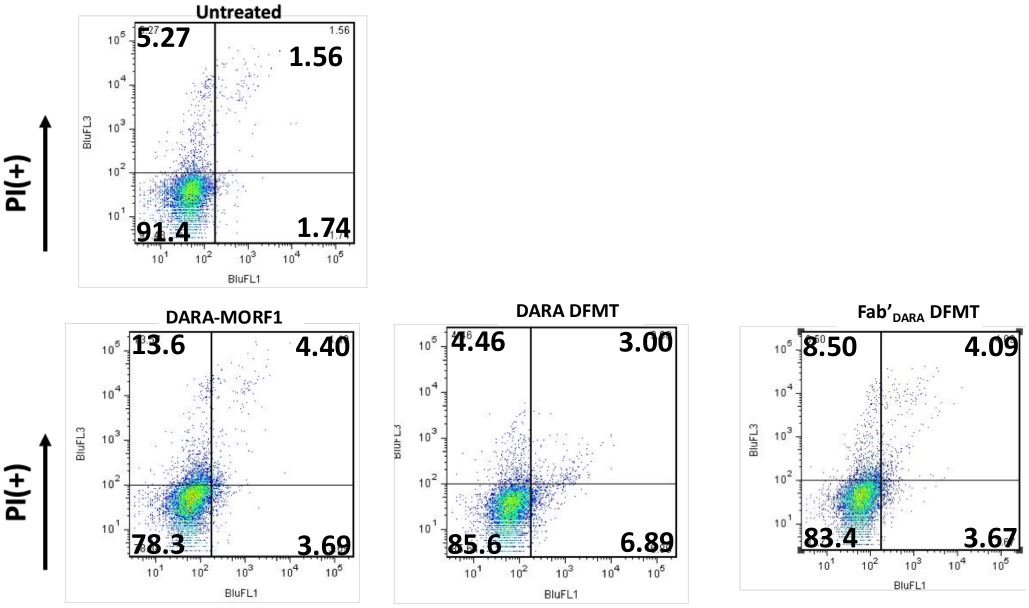

Figure S15. Caspase 3 cell populations post-gating and post-caspase 3 measurement. Propidium iodide (PI) was used to stain the cells after measurements to assess degree of membrane integrity. A high PI signal would indicate leaky cell membranes and unreliable caspase 3 measurements. No significant PI-positive cell population shift was observed. Experiment was performed in triplicate and corresponds to Figure 4C.

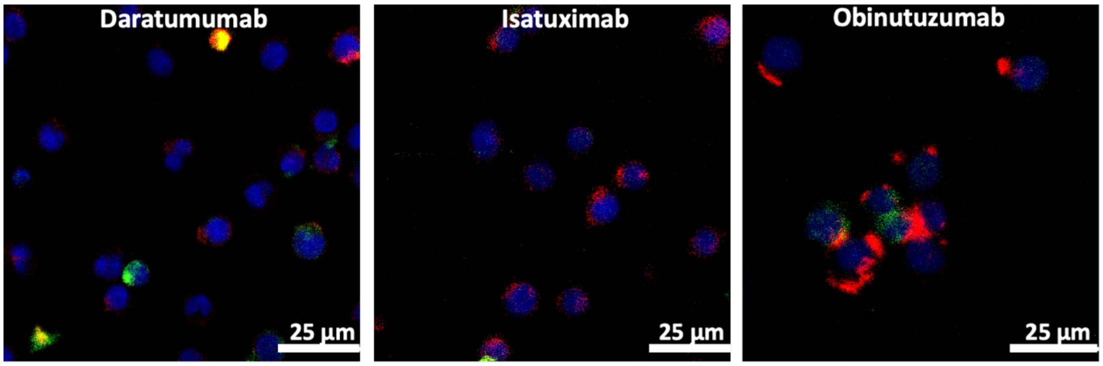

Figure S16. Lipid raft confocal microscopy images of Raji cells treated with naked antibodies. Cells were treated with Cy5-labeled antibodies (red) and subsequently stained with Cholera Toxin Subunit B Alexa Fluor 555 (green) to detect lipid raft redistribution in antibody treated cells. Cell nuclei were stained with Hoechst dye (blue). These images correspond with confocal microscopy of Figure 5C.

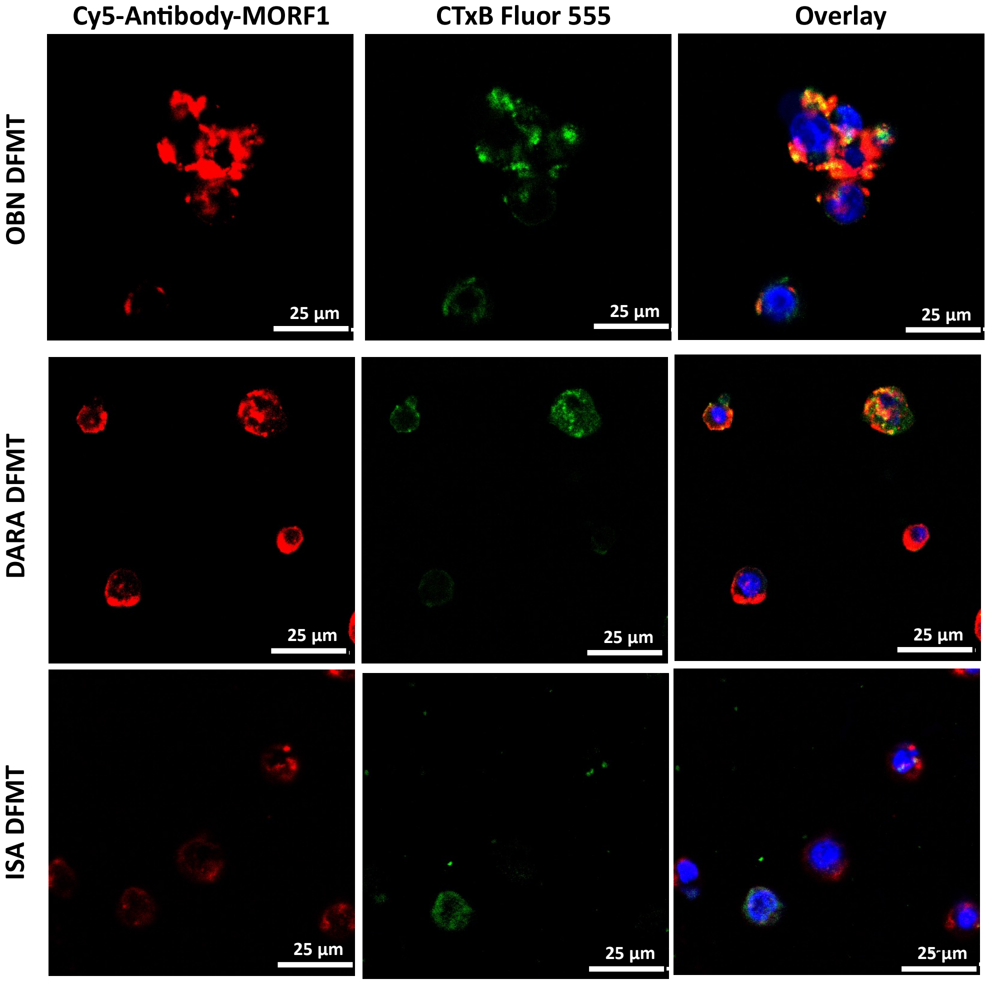

Figure S17. Additional confocal microscopy images of DFMT-induced lipid raft redistribution of Raji cells. OBN DFMT (top row) was compared to DARA DFMT (middle row) and ISA DFMT (bottom row). Cy5-labeled antibody-MORF1 (red) was used in the DFMT. Cells were stained with Cholera Toxin Subunit B Alexa Fluor 555 (green) to detect lipid raft redistribution in DFMT treated cells. Cell nuclei were stained with Hoechst dye (blue).

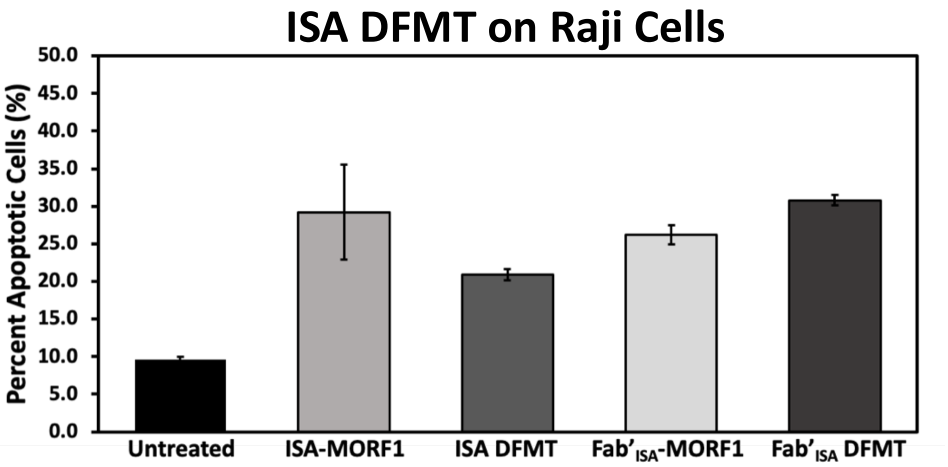

Figure S18. ISA DFMT & Fab'$_{ISA}$ DFMT on Raji cells. Percent apoptotic cells treated with ISA or Fab'$_{ISA}$ DFMT for 24 h. Analyzed with flow cytometry via Annexin V/PI stain. Corresponds with cellular apoptotic data of ISA-treated Daudi cells in Figure 6 of the main text.
